# Plexin-A2 enables the proliferation and the development of tumors from glioblastoma derived cells

**DOI:** 10.1038/s41419-023-05554-0

**Published:** 2023-01-19

**Authors:** Shira Toledano, Adi D. Sabag, Neta Ilan, Tanya Liburkin-Dan, Ofra Kessler, Gera Neufeld

**Affiliations:** 1 Cancer research center, The Bruce Rappaport Faculty of Medicine, Technion-Israel Institute of Technology, Haifa, 3109602 Israel; 2 Division of Allergy & Clinical Immunology, Bnai-Zion medical Center, Haifa, 33394 Israel

**Keywords:** CNS cancer, Cell-cycle exit

## Abstract

The semaphorin guidance factors receptor plexin-A2 transduces sema6A and sema6B signals and may mediate, along with plexin-A4, the anti-angiogenic effects of sema6A. When associated with neuropilins plexin-A2 also transduces the anti-angiogenic signals of sema3B. Here we show that inhibition of plexin-A2 expression in glioblastoma derived cells that express wild type p53 such as U87MG and A172 cells, or in primary human endothelial cells, strongly inhibits cell proliferation. Inhibition of plexin-A2 expression in U87MG cells also results in strong inhibition of their tumor forming ability. Knock-out of the plexin-A2 gene in U87MG cells using CRISPR/Cas9 inhibits cell proliferation which is rescued following plexin-A2 re-expression, or expression of a truncated plexin-A2 lacking its extracellular domain. Inhibition of plexin-A2 expression results in cell cycle arrest at the G2/M stage, and is accompanied by changes in cytoskeletal organization, cell flattening, and enhanced expression of senescence associated β-galactosidase. It is also associated with reduced AKT phosphorylation and enhanced phosphorylation of p38MAPK. We find that the pro-proliferative effects of plexin-A2 are mediated by FARP2 and FYN and by the GTPase activating (GAP) domain located in the intracellular domain of plexin-A2. Point mutations in these locations inhibit the rescue of cell proliferation upon re-expression of the mutated intracellular domain in the knock-out cells. In contrast re-expression of a plexin-A2 cDNA containing a point mutation in the semaphorin binding domain failed to inhibit the rescue. Our results suggest that plexin-A2 may represent a novel target for the development of anti-tumorigenic therapeutics.

## Introduction

Semaphorins were initially characterized as axon guidance factors, but have subsequently been found to regulate angiogenesis, lymphangiogenesis, immune responses and to modulate tumor progression [1, 2]. The nine receptors of the plexin family function as semaphorin receptors [3] and are divided into four subfamilies. They are single-pass membrane receptors that are characterized by the presence of a GTPase activating (GAP) domain in their intracellular domain [4]. Upon activation by semaphorins they induce the local collapse of the cytoskeleton in target cells and thus function mostly as repulsive guidance factors. Most semaphorins bind directly to plexins except for six of the seven class-3 semaphorins which bind to receptors of the neuropilin family. Neuropilins do not transduce semaphorin signals on their own due to their short intracellular domains. Following the binding of the class-3 semaphorins, they associate with type-A plexins or with plexin-D1, thereby activating plexin-mediated signal transduction [1, 5]. Several class-3 semaphorins such as sema3A, sema3B and sema3F have been found to function as potent inhibitors of tumor angiogenesis and consequently as inhibitors of tumor progression [6–10].

Plexin-A2 is one of the four type-A plexins. Neuropilin-1 and neuropilin-2 can associate with this receptor to transduce sema3B anti-angiogenic signals and sema3C signals [7, 11–14]. Plexin-A2 can also form complexes with plexin-A4 and both of these receptors function as direct binding and signal-transducing receptors for sema6A and sema6B [15, 16]. Plexin-A2 as well as plexin-A4 may mediate the anti-angiogenic effects of sema6A [17]. We have previously found that sema6B produced by U87MG glioblastoma-derived cells as well as by human umbilical vein-derived endothelial cells (HUVEC), induces plexin-A4 mediated cell proliferation [18]. High plexin-A2 expression levels were also linked to worse prognosis in glioblastoma and in prostate cancer (Oncomine, Murat brain dataset, (Fig. S1A)) [19–21].

To better understand the involvement of plexin-A2 in glioblastoma we have inhibited the expression of plexin-A2 in U87MG glioblastoma derived cells using several methods, including gene knock-out with CRISPR/Cas9. This resulted in an almost complete inhibition of their proliferation and their tumor-forming ability and was also accompanied by profound changes in their cytoskeletal organization that resembled changes associated with cellular senescence. Similar changes were observed following the silencing of plexin-A2 expression in A172 glioblastoma-derived cells. In contrast, silencing plexin-A2 expression had no effect in glioblastoma-derived cells containing mutations in the p53 gene. The inhibition of cell proliferation as well as the cytoskeletal changes could be rescued by the expression of cDNAs encoding either full-length plexin-A2 or a truncated plexin-A2 lacking the entire extracellular domain. We present evidence suggesting that the pro-proliferative activity of plexin-A2 is not dependent on autocrine activation by sema3C and further show evidence suggesting that the pro-proliferative activities of plexin-A2 require the plexin-A4 receptor and are mediated, at least in part, by the secondary messengers FARP2, FYN, AKT and p38MAPK. Our results suggest that targeting plexin-A2 in glioblastoma that retain wild-type p53 expression, may turn out to have therapeutic value.

## Results

### Silencing plexin-A2 expression in U87MG cells inhibits their proliferation and their tumor-forming ability

**Fig. 1:**
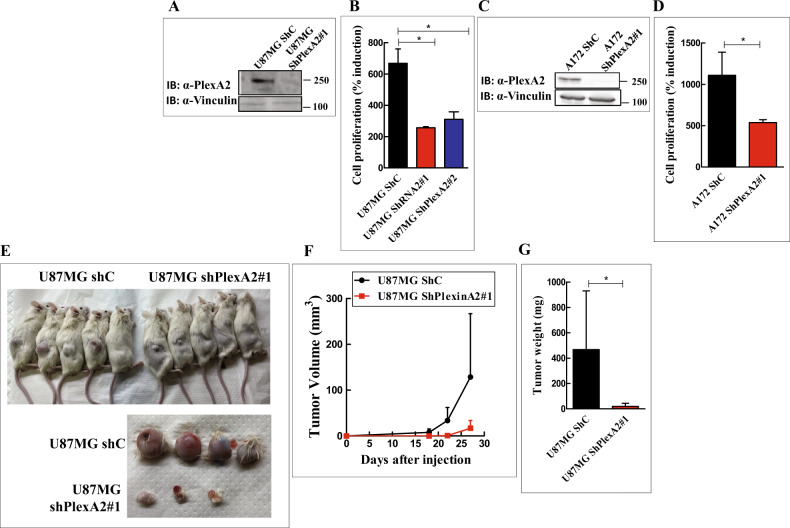
Silencing plexin-A2 expression in U87MG and A172 cells inhibits their proliferation and the development of subcutaneous tumors. **A** Western blot showing plexin-A2 expression levels in U87MG cells expressing a non-specific shRNA (ShC) or a shRNA targeting plexin-A2 (ShPlexA2#1). Cell lysates were probed with antibodies directed against plexin-A2 or vinculin. **B** U87MG or U87MG cells silenced for plexin-A2 expression using two different shRNAs were seeded in triplicate (2 × 10^4^ cells/well) in 24 well dishes. Cells were counted in a coulter counter after 3 days. Shown is the percentage of attached cells on day 3 compared with the number of cells attached on day 0 which was taken as 100%. Data are represented as mean ± SD. Statistical analysis was done using the one tailed Mann-Whitney test. *P < 0.05, *N* = 3. **C** Western blot showing plexin-A2 expression levels in A172 cells expressing a non-specific shRNA (ShC) or a shRNA targeting plexin-A2 (ShPlexA2#1). Cell lysates were probed with antibodies directed against plexin-A2 or vinculin. **D** A172 cells were infected with lentiviruses encoding a control shRNA (ShC) or a shRNA targeting plexinA2 (ShPlexA2#1). Each group of cells was seeded in quadruplicate in 96 well dishes (3 × 10^3^ cells/well). Cell proliferation was measured using the WST-1 proliferation assay as described in materials and methods, and the values presented were calculated as described under (**B**). Data are represented as mean ± SD. Statistical analysis was done using the one tailed Mann-Whitney test. *P < 0.05, *N* = 3. **E** U87MG expressing a nonspecific shRNA (ShC) or U87MG cells silenced for plexin-A2 expression using ShPlexA2#1 were injected subcutaneously into NOD/SCID mice (3 × 10^6^ cells/mouse). After 30 days the experiment was terminated, tumors were excised, weighed and photographed. Each group contained 5 mice. Shown are pictures of the mice (upper panel) and of the excised tumors (lower panel). **F** U87MG expressing non-specific shRNA (ShC) or U87MG cells silenced for plexin-A2 expression using ShPlexA2#1 were injected subcutaneously into Athymic/Nude mice (2 × 10^6^ cells/mouse). Each group contained 7 mice. Tumor development was measured twice a week using calipers. Data are represented as mean ± SD. **G** Shown is the mean weight of 19 tumors excised from three independent experiments done as in (**F**). Data are represented as mean ± SD. Statistical analysis was done using the one tailed Mann-Whitney test. *P < 0.05.

U87MG glioblastoma cells express the four class-A plexins as well as both neuropilin receptors but not the plexin-D1 receptor [11, 13, 18]. We have silenced the expression of plexin-A2 in U87MG and A172 glioblastoma cells as well as in human umbilical vein-derived endothelial cells (HUVEC) using several specific shRNA species. The silencing resulted in strong inhibition of cell proliferation (Fig. 1A–D & S1B). To determine if the decreased proliferation was associated with increased cell death we performed sytox Green exclusion assays. However, we found no indication that the decreased proliferation rate of the silenced cells was associated with increased cell death (Fig. S2). When U87MG cells in which the expression of plexin-A2 was silenced were implemented subcutaneously in immune-deficient mice, the development of tumors from the silenced cells was strongly delayed (Fig. 1E–G). Notably, in one of the experiments we did there was no tumor growth at all from the implanted plexin-A2 silenced cells (Fig. S1F). Surprisingly, the size of cells in tumors that developed from silenced cells appears to be smaller than in control tumors (Fig. S1E). Both the U87MG and A172 cells express wild type p53 [22]. Interestingly, Silencing the expression of plexin-A2 in glioblastoma-derived cell lines expressing mutated P53 such as U373MG, U118MG or T98G cells [22] failed to inhibit cell proliferation (Figs. S1C & S1D) even though these cells do express plexin-A2 receptors (Fig. S1G). Single-pass receptors such as tyrosine-kinase receptors or plexins usually form homo or hetero dimers to transduce signals [11, 18]. To disrupt the hypothetical dimerization-dependent pro-proliferative signaling of plexin-A2, we also expressed in U87MG cells cDNA encoding a truncated dominant-negative plexin-A2 lacking the intracellular domain (A2ExTm) (Fig. S3A). The expression of this cDNA also inhibited cell proliferation suggesting that plexin-A2 homo or hetero dimerization may indeed be required for its pro-proliferative activity (Figs. S3B & S3C).

### Generation of U87MG cells lacking functional plexin-A2 receptors

**Fig. 2:**
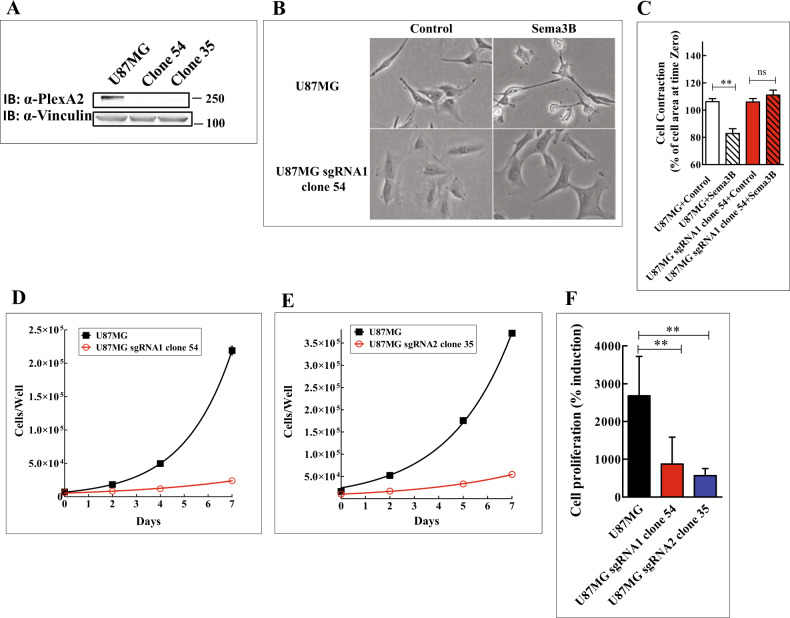
CRISPR/Cas9 mediated knock-out of plexin-A2 expression in U87MG cells inhibits their proliferation. **A** Two different guide RNAs were used to knock-out plexin-A2 expression in U87MG cells. Cell lysates of U87MG cells and knock-out cells (U87MG sgRNA1 clone 54 and U87MG sgRNA2 clone 35) were probed with antibodies directed against plexin-A2 or vinculin. **B** Conditioned medium from HEK293 cells infected with an empty lentiviral expression vector (Control) or from HEK293 cells expressing recombinant sema3B (300 μl) were added to parental U87MG cells or to U87MG sgRNA1 clone 54. Following a 30 min. incubation at 37 ^o^C the cells were photographed. **C** U87MG cells and clone 54 plexin-A2 knock-out cells were photographed at the beginning of the experiment and again 30 min after stimulation with sema3B as described under (**B**) using the Incucyte Zoom HD/2CLR System. Changes in the area of the indicated cell types were determined in fields derived from three replicate wells using the Image-Pro Premier software. Data are represented as mean ± SD. Statistical analysis was done using the one-tailed Mann-Whitney test. *P < 0.05, ns: non-specific, *N* = 4. **D, E** Representative growth curves of U87MG cells and plexin-A2 knock-out clone 54 cells (**D**) and clone 35 cells (**E**). Cells were seeded in quadruplicate (1 × 10^4^ cells/well). Adherent cells were counted every two days using a coulter-counter. Data are represented as mean ± SD. **F** The number of cells attached on day 0 was taken to be 100%. The average percentage of adherent cells on day 7 is shown. Compared were parental U87MG cells (*N* = 13), plexin-A2 clone 54 knock-out cells (*N* = 8) and plexin-A2 clone 35 knock-out cells (*N* = 5). Data are represented as mean ± SD. Statistical analysis was done using the one-tailed Mann-Whitney test. **P < 0.01.

Silencing gene expression using shRNAs or the use of dominant negative inhibitors do not inhibit completely the expression of target genes. To determine the mechanisms by which plexin-A2 affects cell proliferation, we generated U87MG cells in which we have knocked-out the plexin-A2 gene by the introduction of frame-shift mutations into each of the alleles encoding plexin-A2 using CRISPR/Cas9. In order to differentiate as much as possible between true effects of plexin-A2 knock-out and off-target effects we used two different plexin-A2 specific guide RNAs (Fig. S4A) [23]. We have subsequently isolated by limiting dilution several single cell-derived clones of cells, such as clones 35 and 54 that were generated using the different guide RNAs, in which each of the plexin-A2 encoding alleles contained frame shift mutations (Fig. S4B). These two clones no longer expressed plexin-A2 receptors (Fig. 2A) and have lost their ability to respond by cytoskeletal collapse to stimulation with sema3B (Fig. 2B–C) [11]. The proliferation of both knock-out clones was also very strongly inhibited as compared to the proliferation rate of U87MG cells (Fig. 2D–F) further suggesting that plexin-A2 is somehow able to promote the proliferation of U87MG cells.

### The proliferation of plexin-A2 knock-out U87MG cells is rescued following re-expression of the plexin-A2 cDNA

**Fig. 3:**
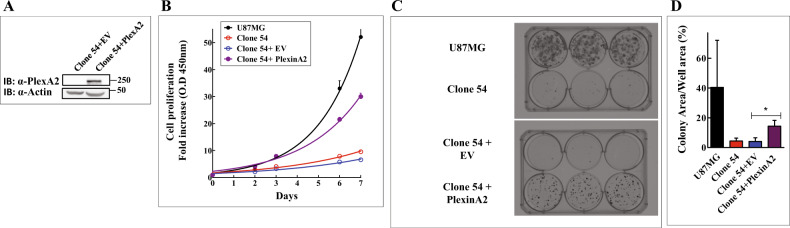
Re-expression of plexin-A2 cDNA in plexin-A2 knock-out cells, restores their proliferation and colony forming ability. **A** U87MG KO clone 54 cells were infected with an empty lentiviral expression vector (EV) or with a lentiviral vector directing expression of plexin-A2 (PlexA2). Western blots prepared from cell lysates were probed with antibodies directed against plexin-A2 and actin. **B** U87MG cells, U87MG clone 54 cell and clone 54 cells infected with empty lentiviruses (EV) or lentiviruses directing expression of plexin-A2 (PlexinA2) were seeded in quadruplicate in 96 well dishes. Cell proliferation was determined using the WST-1 assay as described. Shown is a representative experiment out of *N* = 4 independent experiments. Data are represented as mean ± SD. **C** U87MG cells, U87MG clone 54 cells and U87MG KO clone 54 cells infected with an empty lentiviral expression vector (EV) or with a lentiviral vector directing expression of plexin-A2 (PlexinA2). Cells were seeded in 6 well plates (300 cells/well). After 16 days colonies were fixed in 4% para-formaldehyde and stained with crystal violet. The experiment was repeated three times with similar results. **D** The areas of the colonies formed by the indicated cell types out of the entire well area was determined and is represented as a percentage of the total area. Data are represented as mean ± SD. Statistical analysis was done using the one tailed Mann-Whitney test. *P < 0.05, *N* = 3.

In order to verify that the inhibition of cell proliferation observed following the knock-out of the plexin-A2 gene in U87MG cells is not due to off-target effects, we re-expressed the full-length cDNA encoding plexin-A2 in cells of the U87MG derived plexin-A2 knock-out clone 54 (Fig. 3A). The proliferation of these cells was almost completely rescued following the re-expression of the plexin-A2 cDNA in three separate experiments (Fig. 3B). We also performed colony formation assays using parental U87MG cells, U87MG KO clone 54 cells and U87MG KO clone 54 cells in which we expressed the cDNA encoding the full-length plexin-A2 cDNA. While clone 54 knock-out cells were almost completely unable to form colonies, re-expression of the plexin-A2 cDNA resulted in partial rescue of colony formation ability (Fig. 3C–D).

### Expression of a truncated plexin-A2 lacking the extracellular domain restores cell proliferation of plexin-A2 knock-out cells

**Fig. 4:**
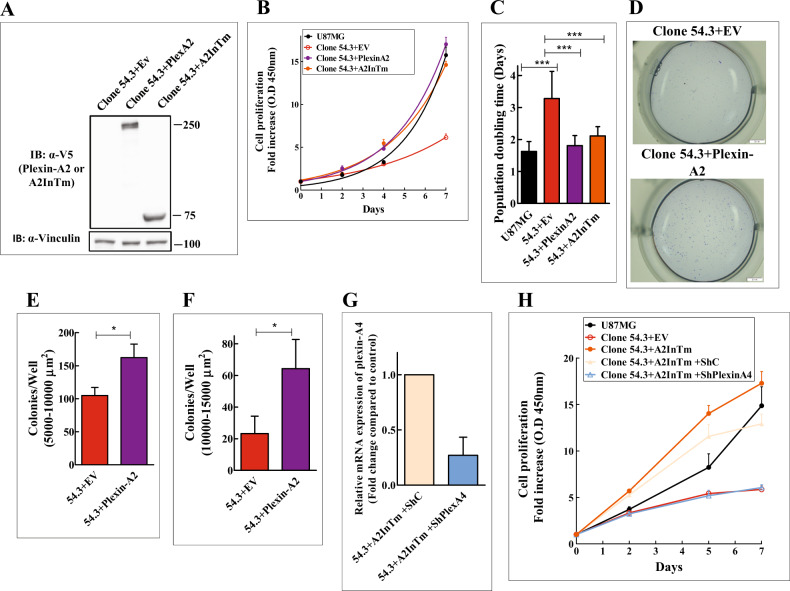
Re-expression of a truncated plexin-A2 containing the intracellular and trans-membrane domains (A2InTm) of plexin-A2 restores cell proliferation of plexin-A2 knock-out cells. **A** Plexin-A2 knock-out clone 54.3 cells were infected with an empty lentiviral expression vector (EV) or a lentiviral vector directing expression of a V5 tagged full length plexin-A2 (PlexA2) or a truncated plexin-A2 containing the intracellular and trans-membrane domains of plexin-A2 (A2InTm). Western blots prepared from cell lysates were probed with antibodies directed against V5 and vinculin. **B** Representative growth curves of U87MG cells and of U87MG KO clone 54.3 cells infected with lentiviruses encoding plexin-A2 (PlexinA2), A2InTm or an empty expression vector (EV). Cells were seeded in quadruplicate in 24 well dishes (1 × 10^4^ cells/well). Cells were counted every two days using a coulter-counter. Data are represented as mean ± SD. **C** The average population doubling time of U87MG cells (*N* = 11) was compared with the average population doubling time of clone 54.3 + EV (*N* = 11), clone 54.3 + PlexinA2 (*N* = 7) and clone 54.3 + A2InTm (*N* = 8). Data are represented as mean ± SD. Statistical analysis was done using the one tailed Mann-Whitney test. ***P < 0.001 **D** Clone 54.3 cells infected with an empty lentiviral expression vector (EV) or with a lentiviral vector directing expression of plexin-A2 were seeded in soft agar in 24 well dishes (1 × 10^3^ cells/well) in triplicates. Colonies were stained with crystal violet after 13 days. Shown are photographs of representative wells. **E**, **F** The average number of colonies per well with an area between 5000 and 10,000 µm^2^ or 10,000 and 15,000 µm^2^ were determined. Data are represented as mean ± SD. Statistical analysis was done using the one-tailed Mann-Whitney test. *P < 0.05, *N* = 3. **G** Lentiviruses were used to express control (ShC) or a plexin-A4 targeting shRNA (ShPlexA4) in KO clone 54.3 cells expressing A2InTm. The expression of plexin-A4 was then examined using qRT-PCR. Data are represented as mean ± SD, *N* = 2. **H** Representative growth curve of U87MG cells and of clone 54.3 cells expressing an empty expression vector (EV) or expressing A2InTm and clone 54.3 cells expressing A2InTm as well as a non-specific shRNA (ShC) or Clone 54.3 cells expressing A2InTm and an shRNA targeting plexin-A4 (ShPlexinA4). The various cells were seeded in quadruplicate in 96 well dishes (3 × 10^3^ cells/well). The proliferation of the cells was determined every two days using the WST-1 assay as previously described. Data are represented as mean ± SD. The experiment was repeated three times with similar results.

Clone 54 and clone 35 plexin-A2 knock-out cells proliferated at an extremely slow rate that made working with them very time-consuming and difficult. We have therefore isolated, by limiting dilution, a subclone of the clone 54 cells which displayed a somewhat faster rate of basal proliferation (clone 54.3). Like clone 54 cells, the proliferation of clone 54.3 cells was also completely rescued following re-expression of the full-length plexin-A2 cDNA (Fig. 4A–C). Expression of plexin-A2 in clone 54.3 cells, also enhanced the growth of anchorage-independent colonies in soft agar, which was strongly suppressed in the knock-out cells (Fig. 4D–F).

To identify plexin-A2 domains responsible for the transduction of its pro-proliferative signals in U87MG cells we first expressed in clone 54.3 cells a truncated plexin-A2 lacking the extracellular domain of plexin-A2 (A2InTm) (Fig. 4A). The expression of A2InTm also rescued efficiently the proliferation of the clone 54.3 cells (Fig. 4B & C), suggesting that elements in the intracellular domain are likely responsible for the transduction of the pro-proliferative activity of plexin-A2.

We have previously observed that silencing the expression of plexin-A4 in U87MG inhibits the proliferation of U87MG cells as well as their tumor forming ability [18]. It was recently observed that plexin-A2 and plexin-A4 form hetero dimers [16]. Taken together these observations suggested that the pro-proliferative effects may be mediated by a plexin-A2/plexin-A4 receptor complex rather than by plexin-A2 alone. Indeed, we found that when we silence the expression of plexin-A4 in clone 54.3 plexin-A2 knock-out cells, the expression of A2InTm alone is no longer sufficient to rescue the proliferation of the cells (Fig. 4G & H).

### knocking-out plexin-A2 in U87MG cells results in cell cycle arrest and in the acquisition of characteristics associated with senescence

**Fig. 5:**
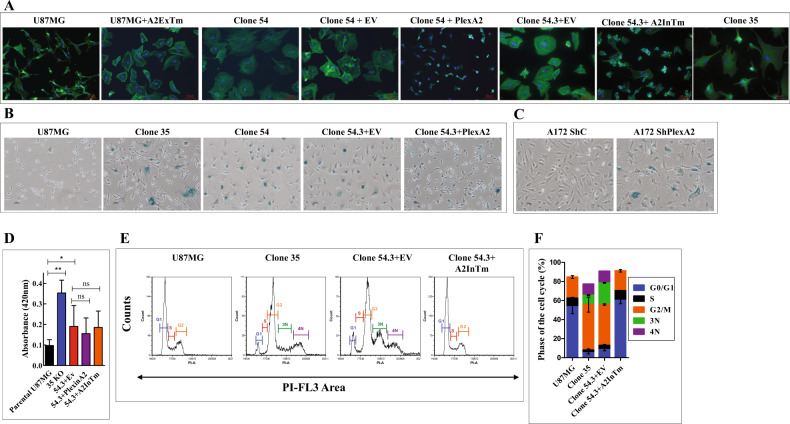
U87MG cells in which the plexin-A2 gene was knocked out acquire some properties of senescent cells. **A** The indicated cell types were seeded on fibronectin-coated coverslips and stained with DAPI to visualize cell nuclei (blue) and with fluorescent phalloidin (green) to visualize actin fibers. Shown are merged confocal photographs generated using ZEN 2.3 software. **B-C** The indicated cell types were assayed at pH-6 for the expression of the senescence marker SA-β galactosidase as described in materials and methods. Shown are representative photographs. **D** Equal numbers of U87MG cells (*N* = 9 independent experiments), plexin-A2 knock out U87MG cells (clone 54.3 (*N* = 7), clone 35 (*N* = 3)) or clone 54.3 cells in which full-length plexin-A2 (PlexinA2) (N = 5) or A2InTm (*N* = 5) were re-expressed, were collected and lysed in 100 µL of 0.1 M phosphate buffer (pH 6.0). β-galactosidase activity was then determined at pH 6.0 as described. Data are represented as mean ± SD. Statistical analysis was done using the one-tailed Mann-Whitney test. *P < 0.05, **P < 0.01, ns: non-specific. **E** Cytofluorimetric analysis of propidium iodide-stained triplicates of U87MG cells, U87MG clone 35 cells and clone 54.3 cells infected with empty lentiviruses (EV) or A2InTm. The peaks represent cells in G0/G1 (G1), Cells in S (S), cells in G2/M (G2), and aneuploid cells (3 N,4 N). **F** The percentage of cells in the different cell cycle phases is shown for each of the cell types shown in (**E**). Data are represented as mean ± SD. *N* = 3.

U87MG cells in which plexin-A2 was knocked-out (clone 54) displayed a flattened morphology and resembled in appearance and proliferation rate senescent cells (Fig. 5A). Similar changes were also observed in clone 35 cells (Fig. 5A). U87MG cells in which we expressed the dominant-negative truncated plexin-A2 lacking the intracellular domain (A2ExTm) likewise have a flattened morphology (Fig. 5A). These morphological changes were completely reversed following re-expression of full-length plexin-A2 or of A2InTm. The cells regained a polarized appearance, contracted, and appeared to be even smaller than parental U87MG cells, possibly because of plexin-A2 over-expression (Fig. 5A). In contrast with wild-type U87MG cells, the knock-out cells stained positive for senescence-associated beta-galactosidase (SA-β-gal) activity (Fig. 5B & D) [24], suggesting that the knock-out cells display at least some characteristics of senescent cells, and further suggesting that plexin-A2 transduces in these cells signals that may inhibit cellular senescence. Similar morphological changes and staining for SA-β-gal activity were observed when the expression of plexin-A2 in U87MG cells or in A172 glioblastoma cells was inhibited using shRNAs targeting plexin-A2 expression (Fig. 5C & S5A). Surprisingly, the SA-β-gal activity was not suppressed following re-expression of plexin-A2 (Fig. 5B & D) or A2InTm (Fig. 5D).

Cell cycle analysis of clone 54.3 knock-out cells revealed a decrease in the fraction of cells found in the G0/G1 and S phases of the cell cycle, which was accompanied by an increase in the percentage of G2 cells as compared with wild-type U87MG cells or with plexin-A2 knock-out cells in which we have expressed A2InTm (Fig. 5E & F). Furthermore, we could detect an increase in the percentage of aneuploid cells in plexin-A2 knock-out cells (Fig. 5E & F). These observations suggest that inhibition of plexin-A2 expression inhibits cell cycle progression in the G2/M phase.

**Fig. 6:**
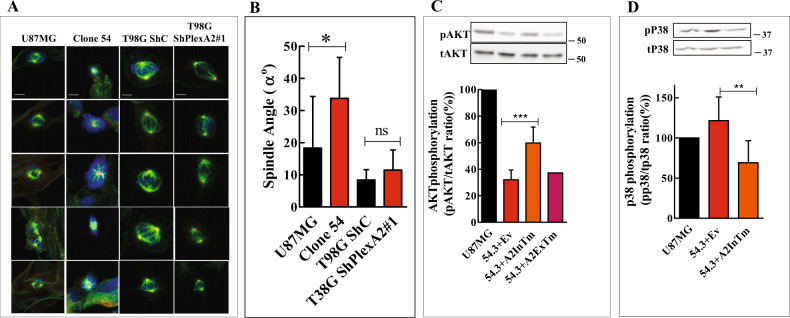
U87MG cells in which the plexin-A2 gene is knocked-out have abnormal mitotic spindles and display changes in the phosphorylation of AKT and P38. **A** The indicated cell types were seeded on fibronectin-coated coverslips and stained with DAPI to visualize cell nuclei (blue) and with antibodies to alpha-tubulin (green) and gamma-tubulin (red). Shown are 3D confocal photographs generated using the Imaris software. Scale bars: 10 μm. **B** The X–Z axis stack projections of the photographs generated from laser-scanning confocal images taken in 0.3 μm steps were quantified using ZEN 2.3 software. The mitotic spindle angles of the indicated cell types were calculated. Data are represented as mean ± SD. Statistical analysis was done using the one-tailed Mann-Whitney test. *P < 0.05, *N* = 10 (U87MG), *N* = 8 (clone 54), *N* = 11 (T98G ShC), *N* = 6 (T98G ShA2). **C** The cDNAs encoding A2InTm, A2ExTM or an empty expression vector (EV) were expressed in clone 54.3 knock-out cells (54.3). The phosphorylation levels of AKT were assayed in the indicated cell types by western blot analysis of cell lysates as described in methods, using an antibody directed against phosphorylated AKT (ser473). Loading was assessed using an antibody directed against total AKT. Shown is a representative western blot. The effect of A2InTm expression on the average phosphorylation levels of AKT was determined (*N* = 8) (except for A2ExTm expressing cells, *N* = 1). Below is shown a histogram depicting the ratio between the intensity of phospho-AKT staining and total AKT. Data are represented as mean ± SD. Statistical analysis was done using the one-tailed Mann-Whitney test. ***P < 0.001, **D** The phosphorylation levels of p38 were assayed in the indicated cell types by western blot analysis of cell lysates as described in methods, using an antibody directed against phosphorylated p38 (Thr180/Tyr182). Loading was assessed using an antibody directed against total p38. Shown is a representative western blot. Below is shown a histogram depicting the average ratio between the intensity of the respective phospho-p38 bands and the total p38 bands. Data are represented as mean ± SD. Statistical analysis was done using the one-tailed Mann-Whitney test. **P < 0.01, *N* = 6.

Although the knock-out of plexin-A2 strongly inhibits the proliferation of the cells some dividing cells could still be detected in clone-54 cultures. However staining with gamma and alpha-tubulin and with DAPI revealed major changes in the mitotic spindle organization in the knock-out cells. The separation of the spindle poles was small as compared with the parental U87MG cells. The microtubuli that connect the two poles were not detectable but instead the cells contained prominent bundles of astral-like alpha tubulin radiating towards the periphery of the cells. Such bundles were not evident in parental U87MG cells. Finally, while in the U87MG cells DAPI staining was confined to cromosomes located between the two spindle poles, in the silenced cells dapi staining surrounded the mitotic spindle. In contrast, no such changes were detected in T98G cells that express mutated p53 (Fig. 6A and S5B). In addition we found that the knock-out changes the angle of the spindle poles as compared with their orientation in parental U87MG cell. In contrast, no such change was observed in T98G cells in which plexin-A2 expression was silenced (Fig. 6B).

### Inhibition of plexin-A2 expression is accompanied by decreased AKT phosphorylation and by enhanced phosphorylation of p38-MAPK

To identify secondary messengers that transduce the pro-proliferative signals of plexin-A2, we screened for known secondary messengers whose phosphorylation state changes following plexin-A2 knock-out in U87MG cells. These experiments revealed that the phosphorylation of AKT on ser473 is inhibited in plexin-A2 clone 54.3 knock-out cells (Fig. 6C) as well as in clone 35 knock-out cells (Fig. S5C). In contrast the phosphorylation of p38-MAPK was induced on Tyr182 in these cells (Fig. 6D & S5D). The changes we observed in the phosphorylation states of p38 and AKT are specific to plexin-A2 since AKT phosphorylation is restored following expression of A2InTm in clone 54.3 knock-out cells while the phosphorylation of p38 is reduced to basal levels (Fig. 6C & D). The restoration is not due to an artifact caused by infection stress since expression of an empty vector (Fig. 6C & D) or of the dominant negative form of plexin-A2, which lacks the intracellular domain (Fig. 6C) did not reverse the phosphorylation state to pre-knock-out levels.

### The FARP2 binding site and the FYN-dependent phosphorylation sites of plexin-A2 mediate the pro-proliferative activity of plexin-A2

To identify plexin-A2 intracellular sub-domains that mediate the pro-survival/pro-proliferative activity of plexin-A2, we introduced point mutations into several known functional sub-domains in the plexin-A2 intracellular domain. These included the Rho GTPase binding domain (RBD domain) [25], the KRK motif of the FERM domain that participates in the binding of FARP2 [26], the tyrosine residues that are phosphorylated by the FYN tyrosine-kinase [27], the juxtamembrane cytosolic dimerization interface domain that was found to contribute to plexin-A3 homo-dimerization [28], and into the conserved catalytic arginine residues of the plexin-A2 GAP domain (Fig. S6A and S6C).

**Fig. 7:**
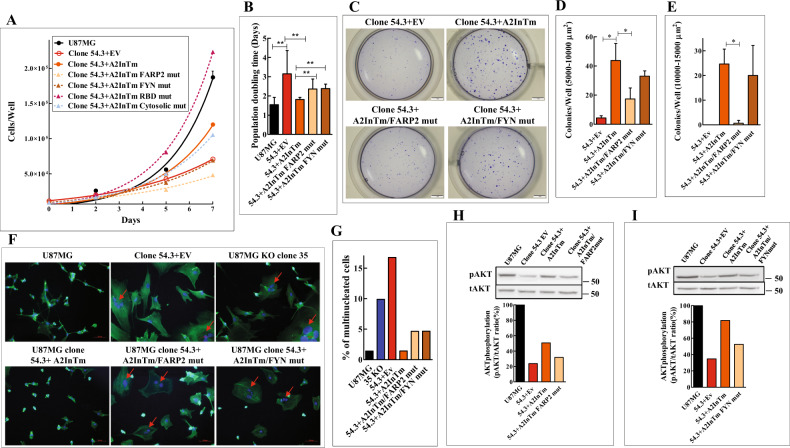
Point mutations in the FARP2 binding site and in the Fyn phosphorylation sites of the plexin-A2 intracellular domain inhibit restoration of cell proliferation induced by A2InTm and inhibit the A2InTm mediated rescue of AKT phosphorylation. **A** U87MG cells, U87MG clone 54.3 cells infected with either control lentiviruses (EV) or with lentiviruses encoding A2InTm or A2InTm variants containing point mutations in the FARP2 binding site (54.3 + A2InTm FARP2 mut), in the Fyn phosphorylation sites (54.3 + A2InTm FYN mut), in the Rho binding domain (RBD) (54.3 + A2InTm RBD mut) or in the juxtamembrane cytosolic dimerization interface (54.3 + A2InTm Cytosolic mut) of plexin-A2 were seeded in 24 well dishes (1 × 10^4^ cells/well). Triplicate wells were counted every two days using a coulter-counter. Data are represented as mean ± SD. Shown is a representative experiment out of five similar experiments. **B** Shown are the average population doubling times of the clone 54.3 derived indicated cell types. Results were derived from *N* = 5 independent experiments. Data are represented as mean ± SD. Statistical analysis was done using the one-tailed Mann-Whitney test. *P < 0.05. **C** Single-cell suspensions of clone 54.3+EV, clone 54.3 + A2InTm, clone 54.3 + A2InTm FARP2 mut or clone 54.3 + A2InTm FYN mut cells, were seeded in soft agar (1 × 10^3^ cells/well) in triplicates. After 17 days colonies of cells were stained with crystal violet. Shown are photographs of representative wells. **D, E** The average number of colonies per well with an area between 5000 and 10,000 µm^2^ or 10,000 and 15,000 µm^2^ was determined. Data are represented as mean ± SD. Statistical analysis was done using the one-tailed Mann-Whitney test. *P < 0.05, *N* = 3. **F** U87MG cells, clone 54.3+EV cells, clone 35 cells, clone 54.3 + A2InTm cells, clone 54.3 + A2InTm FARP2 mut cells and clone 54.3 + A2InTm FYN mut cells were seeded on fibronectin-coated coverslips and stained with DAPI to visualize cell nuclei (blue) and with fluorescent phalloidin (green) to visualize actin fibers. Shown are merged confocal photographs generated using ZEN 2.3 software. Red arrows point at multinucleated cells. **G** The percentage of multinucleated cells of the various cell types described in (**F**) was determined by counting 23 microscopic fields per each cell type. **H** An empty lentiviral expression vector (EV) or lentiviruses expressing cDNAs encoding A2InTm or A2InTm variant containing point mutations in the FARP2 binding site (A2InTm FARP2 mut) were expressed in clone 54.3 knock-out cells (54.3). The phosphorylation levels of AKT were assayed in the indicated cell types by western blot analysis of cell lysates as described in methods. Below is shown a histogram depicting the ratio between the intensity of the respective phospho-AKT bands and the total AKT bands. **I** An empty lentiviral expression vector (EV) or lentiviruses expressing cDNAs encoding A2InTm or A2InTm variant containing point mutations in the Fyn phosphorylation sites (A2InTm FYN mut) were expressed in clone 54.3 knock-out cells (54.3). The levels of AKT phosphorylation were determined as described under (**H**). Below is shown a histogram depicting the ratio between the intensity of the respective phospho-AKT bands and the total AKT bands.

The cDNAs encoding the mutated A2InTm variants were expressed in clone 54.3 knock-out cells (Fig. S6B) and assayed for their ability to rescue cell proliferation. The mutations that were introduced into the RBD domain and into the juxtamembrane cytosolic dimerization interface did not inhibit the rescuing ability of A2InTm (Fig. 7A). In contrast, the mutations introduced into the FARP2 binding site and into the FYN phosphorylation sites each partially inhibited the rescue of cell proliferation by A2InTm (Fig. 7A & B). The introduction of mutations into the FARP2 binding site also inhibited strongly A2InTm’s ability to rescue the formation of anchorage-independent colonies from single cells in soft agar. In contrast, the formation of colonies in soft agar from cells expressing A2InTm containing the FYN phosphorylation sites mutations was not inhibited significantly although a small partial effect that did not reach statistical significance could still be observed (Fig. 7C–E).

A2InTm containing mutations in the FARP2 binding site as well as A2InTm containing mutations in the FYN phosphorylation sites also lost the ability to reverse the morphological changes observed in plexin-A2 knock-out cells (Fig. 7F). We have also found that plexin-A2 knock-out cells contain a higher percentage of multinucleated cells. The concentration of multinucleated cells was strongly reduced following the expression of A2InTm in the knock-out cells. Expression of A2InTm mutated in the FARP2 binding site or in the FYN phosphorylation sites each resulted in a partial decrease in the percentage of multinucleated cells, which was less efficient than the rescue obtained with wild type A2InTm (Fig. 7G) suggesting that the function of both sites is probably required for complete rescue.

### The FARP2 binding site and the FYN phosphorylation sites of plexin-A2 are required for the phosphorylation of AKT

The phosphorylation of AKT on ser473 is inhibited in plexin-A2 knock-out U87MG cells and rescued by A2InTm expression (Fig. 6C). Interestingly, mutations in the FARP2 binding site or in the FYN phosphorylation sites inhibited partially the rescue of AKT phosphorylation suggesting that AKT may indeed mediate, at least in part, the transduction of the pro-proliferative effects of plexin-A2 that are initiated as a result of the interactions of FYN and FARP2 with plexin-A2 (Fig. 7H & I). Indeed, FYN was previously reported to activate AKT to promote glioma cell proliferation [29].

**Fig. 8:**
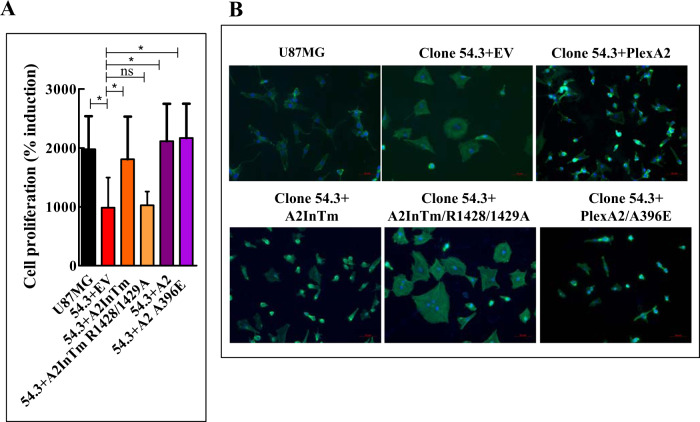
Point mutations in the GAP domain of plexin-A2 inhibit rescue of cell proliferation by the plexin-A2 intracellular domain while a point mutation at the semaphorin binding site of plexin-A2 has no effect. **A** U87MG cells, or U87MG clone 54.3 cells infected with either control lentiviruses (EV) or with lentiviruses encoding A2InTm or A2InTm variants containing point mutations in the conserved catalytic arginine residues of the GAP domain (54.3 + A2InTm R1428/1429 A) or the semaphorin binding site located in the extracellular domain of plexin-A2 (54.3 + A2 A396E), were seeded in quadruplicates in 96 well dishes (3 × 10^3^ cells/well). Cell proliferation was measured using the WST-1 proliferation assay as described in materials and methods. Induction of cell proliferation was calculated as described under (1B). Data are represented as mean ± SD. Statistical analysis was done using the one-tailed Mann-Whitney test. *P < 0.05, ns: non-specific *N* = 5. **B** The indicated cell types were seeded on fibronectin-coated coverslips and stained with DAPI to visualize cell nuclei (blue) and with fluorescent phalloidin (green) to visualize actin fibers. Shown are merged confocal photographs generated using ZEN 2.3 software.

The rescue of cell proliferation by the intra-cellular domain of plexin-A2 was also completely inhibited following the introduction of a point mutation into the conserved catalytic arginine residues of the GAP domain of plexin-A2 (R1428A\R1429A) (Fig. S6C & S6E) and the cells displayed a flattened morphology (Fig. 8a & 8B) [30]. In contrast, re-expression of a plexin-A2 cDNA mutated at the semaphorin binding site (A396E) [31] (Fig. S6C & S6D), which completely inhibited the binding of sema6A to plexin-A2 (Fig. S7A & S7B), did not inhibit cell proliferation rescue and the cells regained a polarized appearance (Fig. 8A & B). It therefore seems that the anti-proliferative effect induced by the silencing of plexin-A2 expression is not the result of disrupted plexin-A2-mediated semaphorin signal transduction.

### The plexin-A2 mediated proliferation of U87MG glioblastoma cells is independent of sema3C

It was reported that inhibition of sema3C expression in glioblastoma cancer stem cells inhibits cell proliferation and it was suggested that this activity is mediated by the plexin-D1 or the plexin-A2 receptors [19]. However, we could not detect any sema3C in the conditioned medium of U87MG cells (Fig. S8A). Sema3E signals exclusively using the plexin-D1 receptor [32]. U87MG cells do not express plexin-D1 receptors (Fig. S1G) [13] and did not respond to stimulation with sema3E by cell contraction (Fig. S8B) [9, 13]. Activation of plexin-A2 mediated signal transduction by sema3C would require the binding of sema3C to the neuropilin-1 or neuropilin-2 receptors, which then associate with plexin-A2 to initiate signal transduction [5, 13, 33]. However, the proliferation of U87MG cells in which we have knocked-out the genes encoding neuropilin-1 and neuropilin-2 using CRISPR/Cas9 [13] is not inhibited (Figs. S8C & S8D) suggesting that the inhibition of cell proliferation induced by the knock-out of plexin-A2 is not due to the disruption of sema3C induced autocrine signaling.

## Discussion

Plexins such as plexin-B1, plexin-B2 and plexin-A4 were recently found to function as transducers of pro-tumorigenic and pro-proliferative signals conveyed by semaphorins such as, sema4D, sema4C and sema6B [18, 34, 35]. Plexin-A2 was previously reported to convey anti-angiogenic signals induced by sema3B [7] and to be downregulated in tumors from several types of cancer [36, 37]. Here we have found that silencing the expression of the plexin-A2 receptor in glioblastoma cells that contain intact p53 using specific shRNA species resulted in strong inhibition of cell proliferation and delayed tumor development. In the small tumors that did develop from the plexin-A2 silenced cells we still detected human plexin-A2 mRNA expression suggesting that there was either spontaneous plexin-A2 re-expression in spite of the silencing or that the silencing was not as efficient in all of the implanted cells causing delayed tumor development. In contrast, silencing plexin-A2 expression in glioblastoma-derived cells which contain mutated p53 [22] had no effect on their proliferation. Mutations in p53 were found to enhance the progression of glioblastoma [38] and may thus mask the effect of plexin-A2 inhibition on cell proliferation.

In order to gain a better understanding of the molecular mechanisms by which plexin-A2 promotes cell proliferation, we used U87MG single cell-derived clones in which we have knocked-out the plexin-A2 gene using CRISPR/Cas9. The proliferation of these knock-out cells was extremely slow, and the clones tended to stop proliferating completely after a while. The plexin-A2 knock-out cells adopted a flattened morphology reminiscent of the shape changes associated with senescent cells. In contrast with parental U87MG cells, the knock-out cells also expressed senescence-associated beta-galactosidase (SA-β-gal) activity [24]. Unexpectedly, the SA-β-gal activity persisted in rescued cells in which plexin-A2 was re-expressed even though the proliferation of the rescued cells was restored and they did not resemble senescent cells anymore. However, similar observations had been reported in the past [39, 40].

Sema6A, sema6B and sema3B transduce their signals using plexin-A2 and plexin-A4 receptors [11, 41]. When the expression of sema6B is inhibited in endothelial cells the cells flatten, and their proliferation is inhibited in a manner reminiscent of the changes observed when plexin-A2 expression is inhibited in U87MG cells [18]. Using U87MG knock-out cells, we found that the proliferation, as well as the morphological changes can be restored to wild-type levels following re-expression of the full-length cDNA encoding plexin-A2 or by the plexin-A2 deletion mutant A2InTm, which lacks the entire extracellular domain. Furthermore, while the knock-out cells contained an elevated concentration of aneuploid cells, no such cells could be detected in the rescued cell population.

It is unclear whether plexin-A2 signaling promotes cell proliferation instructively or only supplies a permissive signal that allows cell proliferation and protects against senescence. These questions will need to be further investigated in the future. However, our experiments clearly suggested that domains in the intracellular part of plexin-A2 are responsible for the pro-proliferative function of plexin-A2. Indeed, point mutations introduced into the FARP2 binding domain of plexin-A2 [26] or point mutations introduced into the two FYN phosphorylation sites of the intracellular domain [27] inhibited each partially the rescue of cell proliferation by A2InTm expression as well as the reversal of the morphological changes, suggesting that both sites are probably required for the full pro-proliferative function of plexin-A2.

To identify potential secondary messengers that convey the pro-proliferative signals of plexin-A2, we screened for secondary messengers whose phosphorylation state is altered in the knock-out cells. We found that the phosphorylation of p38-MAPK was enhanced in the knock-out cells and was rescued following A2InTm expression. In-contrast, the phosphorylation of AKT was inhibited in the plexin-A2 knock-out cells and rescued following A2InTm expression in the cells but was only partially successful when the rescue was done using A2InTm containing mutations in the FARP2 binding domain or in the FYN phosphorylation sites of plexin-A2. Increased phosphorylation of p38MAPK was previously reported to be associated with Ras-induced premature senescence [42] and to independently contribute to senescence [43], while inhibition of AKT was linked to inhibition of glioblastoma progression [44]. It is thus possible that these two secondary messengers play a part in the conveyance of plexin-A2-induced pro-proliferative effects.

The pro-proliferative/pro-survival function of plexin-A2 may be independent of external activation or be activated by an autocrine factor that is constitutively expressed in these cells. However, if the pro-proliferative function is activated by an external factor it is unlikely to be a semaphorin binding to the semaphorin binding site of plexin-A2 since a point mutation in the semaphorin binding site that inhibits completely the binding of the plexin-A2 ligand sema6A failed to inhibit the rescue of cell proliferation by the mutated plexin-A2. It was reported that inhibition of sema3C in glioblastoma cancer stem cells also inhibits cell proliferation and that this is mediated by the plexin-D1 and by the plexin-A2 receptors [19]. However, U87MG cells do not express the sema3C receptor plexin-D1 and therefore fail to respond to stimulation with sema3E, which signals exclusively using plexin-D1 [11, 32]. Even overexpression of plexin-A2 in U87MG cells does not enable sema3C signal transduction [13]. Thus, it seems that the pro-proliferative activity of plexin-A2 is not induced by autocrine sema3C. It is possible that other semaphorins, such as sema6B or some other autocrine factor may activate for the pro-proliferative function of plexin-A2. We have found previously that plexin-A4 silencing also inhibits the proliferation of U87MG cells and tumor formation from these cells [18]. Indeed, when we silenced the expression of plexin-A4 in plexin-A2 knock-out cells, it inhibited completely the rescue of cell proliferation following A2InTm expression. It therefore seems that the transduction of the pro-proliferative signal of plexin-A2 also requires plexin-A4. Indeed, plexin-A2 was found to associate with plexin-A4 in a recent publication [16]. We have also previously found that silencing the expression of the plexin-A2/plexin-A4 ligand sema6B in U87MG cells results in the inhibition of proliferation and tumor formation [18]. It is therefore possible that autocrine sema6B activates a pro-proliferative/pro-survival signal that is mediated by a hetero-dimeric complex of plexin-A2 and plexin-A4, and by secondary mediators such as FARP2, FYN, AKT and p38MAPK. These possibilities will need to be further investigated in the future. Our results suggest a novel role for plexin-A2 as a modulator of glioblastoma cells proliferation and indicate that inhibitors of plexin-A2/plexin-A4 may perhaps represent new targets for the development of anti-tumorigenic drugs.

## Materials and methods

### Antibodies and reagents

Mouse sema3E/FC was produced and purified as previously described [9]. Rabbit anti plexin-A2: Abcam (Cambridge, United Kingdom) (ab39357), Rabbit anti plexin-A4: Sigma-Aldrich (St Louis, MO, USA) (HPA029919), Goat anti plexin-D1: Abcam (ab28762), Mouse anti-vinculin: Chemicon (Darmstadt, Germany) (3574), Goat anti plexin-A2-APC: RαD systems (Minneapolis, MN, USA) (FAB5486A), Alexa Fluor 488 Phalloidin: Invitrogen-Molecular Probes (Waltham, MA, USA) (A12379), Mouse anti-V5: Invitrogen (R960-25), Rabbit anti alpha-tubulin: Abcam (ab4074), mouse anti gamma-tubulin: Sigma-Aldrich (T6557), Rabbit anti phospho-AKT (ser473): Cell Signaling (Danvers, MA,USA) (4060), Mouse anti AKT: Santa-Cruz (Dallas, Texas, USA) (sc-5298), Rabbit anti phospho-p38 MAPK (Thr180/Tyr182): Cell Signaling (#9211), Rabbit anti p38-MAPK: Cell Signaling (#9212), Mouse anti-FLAG: Sigma-Aldrich (F1804),Goat anti mouse IgG H&L (Alexa Fluor®647): abcam (ab150115), Goat anti human IgG Alkaline phosphatase (AP)-conjugated antibody: Jackson ImmunoResearch Laboratories (West Grove, PA, USA) (109-055-098), Goat anti sema3C: Santa-Cruz (sc-27796). NucleoSpin RNA Plus was from Macherey-Nagel (Düren, Germany) (Cat. No. 740984.50). The qScript cDNA Synthesis Kit was from Quantabio (Beverly, MA, USA) (Cat. No. 95047-100). The WST-1 kit was from Roche Diagnostics (Mannheim, Germany) (Cat. No. 05 015 944 001). The Senescence β-Galactosidase staining kit was from Cell Signaling technology (Cat. No. 9860) and Lipofectamine 3000 was purchased from Invitrogen (Cat. No. L3000008). The BCIP/NBT liquid substate was from Sigma-Aldrich (B1911).

### Plasmids

The NSPI-CMV-MCS-myc-His lentiviral expression vector was kindly provided by Dr. Gal Akiri (Mount Sinai School of Medicine, NY) [45]. The plexin-A2/myc plasmid was kindly provided by Dr. Oded Behar (Hebrew University, Jerusalem, Israel). The pDonor221 and pLenti6/V5-DEST plasmids were purchased from Invitrogen. The pENTR1A-GFP-N2 (#19364) plasmid was from Addgene (Watertown, MA, USA) [deposited by Eric Campeau [46]]. The pSpCas9(BB)-2A-GFP (#48138) plasmid was also from Addgene [deposited by Feng Zhang [23]]. The ∆NRF (pCMV dR 8.74) and pMD2-VSV-G vectors for lentivirus production were kindly provided by Dr. Tal Kafri (University of North Carolina at Chapel Hill, NC, USA). The various lentiviral shRNA expression vectors were purchased from Sigma Aldrich. The plexin-A2 cDNA variant containing a double point mutant at the FYN-dependent phosphorylation sites of plexin-A2 (Y1605F/Y1677F) was kindly provided by Dr. Riley M. St. Clair (University of Vermont, Burlington, VT) [27]. A plasmid containing the extra-cellular region of Sema6a fused to the human Fc was kindly provided by Dr. Avraham Yaron (Weizmann Institute of Science, Rehovot, Israel).

### Cell lines

HUVEC were isolated and cultured as previously described [47]. HEK293, U87MG, U373MG, A172, T98G and U118MG cells were purchased from the American Type Culture Collection (ATCC) (Manassas, VA, USA) and cultured as previously described [6, 11, 48]. U87MG, U373MG, A172, T98G and U118MG cells were authenticated in June 2022, and confirmed to be free of mycoplasma contamination. HEK293-FT cells were purchased from Invitrogen. A172 and T98G cells were cultured as previously described for U373 cells. U118MG cells were cultured as previously described for HEK293 cells, the medium was also supplemented with 0.1 mM non-essential amino acids and 1 mM sodium pyruvate (Biological Industries, Beit-Ha’Emek, Israel). Puromycin, 2 µg/ml (Sigma-Aldrich) or blasticidin, 20 µg/ml (Invitrogen) were used to select infected cells.

### Inhibition of plexin expression with shRNA-expressing lentiviruses

Mission plasmids directing expression of shRNAs targeting PlexinA2 (TRCN0000061499 (ShPlexA2#1) and TRCN0000061501 (ShPlexA2#2)) or PlexinA4 (TRCN0000078683) were purchased from Sigma Aldrich. The production of the lentiviruses, infection of cells and the selection of shRNA expression cells in HEK293 cells were performed as described previously [7].

### Generation of plexin-A2 knock-out cells using CRISPR/Cas9

U87MG cells were transfected with a pSpCas9(BB)-2A-GFP plasmids containing two different single guide RNAs (sgRNAs) sequences for plexin-A2 (Fig. S4A). The sgRNAs were chosen by using the CRISPR Design Tool (http://crispr.mit.edu/). Fluorescence-activated cell sorting (FACS) was performed 48 h after the transfection, for the selection of the GFP-expressing cells. The sorted cells were submitted to limiting dilution cloning. Clones containing mutations in the areas adjacent to the sgRNA target area were identified by PCR and the areas adjacent to the DNA region containing the sgRNA target sequence were then sequenced. The DNA sequences obtained were compared to the consensus DNA sequence of the gene and thoroughly examined in order to find the ones with insertion/deletion mutations causing frameshift disruption in both alleles [49] using an online tool (http://shinyapps.datacurators.nl/tide/) [50, 51]. Clones which were found to have null mutations in both alleles were submitted to western blot analysis for confirmation of the protein absence and later for cytoskeleton collapse experiments with sema3B.

### Quantitative real-time PCR

Quantitative real-time PCR was performed using a StepOne Plus Real-Time PCR System (Applied biosystems, Waltham, MA, USA) with TaqMen Universal PCR Master Mix, according to the instructions of the manufacturer (Applied biosystems). The normalizing gene was RPLPO. Data was analyzed by the StepOne Software (Applied biosystems) using the relative Quantitation-Comparative C_T_ method. The following primers were used: PlexinA2- Hs00300697, PlexinA4-Hs00297356 and RPLPO- Hs99999902.

### Expression and production of semaphorins

Class 3 semaphorin cDNAs were subcloned into the NSPI-CMV-myc-his lentiviral expression vector as described [7, 52]. The production of lentiviruses and the generation of conditioned media containing various semaphorins were performed as described [52].

### Cytoskeletal collapse experiments

Cytoskeleton collapse assays using U87MG cells were performed as previously described [11, 18] using HEK293 cell-derived conditioned medium containing recombinant sema3B or control conditioned medium from cells containing empty expression vectors. To stabilize pH, HEPES buffer (10 mM, pH 7.2) was added. Cells were photographed after a 30 min incubation in a humidified incubator at 37 °C using a phase-contrast inverted microscope (Eclipse TE2000-U; Nikon, Tokyo, Japan). Equipped with a digital photomicrographic camera (DXM1200C; Nikon) at 20x magnification. The images were acquired with ACT-1C software (Nikon).

### Expression of recombinant plexins

The full-length cDNA of plexin-A2 was cloned into the gateway pDonor221 plasmid and then transferred by recombination into the pLenti6/V5-DEST lentiviral expression vector in frame with a C-terminal V5 tag according to the instructions of the manufacturer (Invitrogen), as previously described [11]. A deletion mutant of plexin-A2 containing the extracellular and transmembrane domains (amino acids 1–1323) of the protein fused to a C-terminal FLAG tag (A2ExTm) was cloned into the gateway entry vector, pENTR1A-GFP-N2, and then transferred by recombination into the pLenti6/V5-DEST lentiviral expression vector. A deletion mutant of plexin-A2 containing the intracellular and transmembrane domains amino acids (1214–1894) of the protein (A2InTm) was generated by truncation of bases 3640–5685 of the plexin-A2 cDNA. The truncated plexin-A2 cDNA was then assembled using NEBuilder HiFi DNA Assembly Master Mix, according to the instructions of the manufacturer (New England Biolabs, Ipswich, MA, USA) into the NSPI-CMV-MCS-myc-His lentiviral expression vector in frame with an N-terminal signal peptide of human plexin-A4 and a V5 tag. A2InTm was subsequently subcloned similarly into the pLenti6/V5-DEST lentiviral expression vector. Introduction of point mutations into the cDNA of A2InTm at the Rho GTPase binding domain (RBD domain) was generated by replacement of the LVP motif with GGA following a previous publication (Fig. S6A) [25]. Introduction of point mutations into the cDNA of A2InTm at the FERM domain binding site was generated by the replacement of the KRK motif with a triplet of alanine following a previous publication [26] (Fig. S6A). Both of these mutants of A2InTm were similarly cloned into the pLenti6/V5-DEST lentiviral expression vector. We also received a plexin-A2 cDNA variant containing a double point mutant at the FYN-dependent phosphorylation sites of plexin-A2 (Y1605F/Y1677F) which was kindly provided by Dr. Riley M. St. Clair (University of Vermont, Burlington, VT) [27]. We then generated A2InTm mutated at these FYN-dependent phosphorylation sites by truncation of bases 3640-5685 of the plexin-A2 cDNA variant and similarly cloning it into the pLenti6/V5-DEST lentiviral expression vector. Additionally, introduction of point mutations into the cDNA of A2InTm at the cytosolic juxtamembrane dimerization interface was generated by replacement of methionine with phenylalanine following a previous publication [28] (Fig. S6A) and similarly cloned into the pLenti6/V5-DEST lentiviral expression vector. Point mutations in the conserved catalytic arginine residues of the GAP domain were generated by replacement of two arginine with alanine following a previous publication [30] (Fig. S6C) and the mutated cDNA was then cloned into the pLenti6/V5-DEST lentiviral expression vector. The point mutation introduced into the semaphorin domain of plexin-A2 was generated by replacement of an alanine residue with a glutamic acid residue following a previous publication [31] (Fig. S6C) and the mutated cDNA was then cloned into the pLenti6/V5-DEST lentiviral expression vector. Production of lentiviruses using these plasmids and stable infection of target cells were performed essentially as previously described [7]. Target cells expressing plexin-A2 or a deletion mutant of plexin-A2 containing the extracellular and transmembrane domains, were isolated from the pool of the infected cells using FACS. U87MG KO clone 54 cells in which we expressed the cDNA encoding the full-length plexin-A2 (2^.^10^7^ cells) or U87MG cells in which we expressed cDNA encoding a dominant-negative truncated plexin-A2 lacking the intracellular domain of plexin-A2 (1^.^10^7^ cells) were washed with PBS and trypsinized, then resuspended in phosphate-buffer saline (PBS) with 0.2% bovine serum albumin (BSA) and with 10 mM EDTA then incubated with Goat anti plexin-A2-APC (RαD systems) for 40 min in the dark at 25 °C for staining plexin-A2 or with Mouse anti-FLAG (Sigma) for 40 min at 4 °C followed by Goat anti mouse IgG H&L (Alexa Fluor®647) (abcam) for 30 min in the dark at 4 °C for staining dominant-negative truncated plexin-A2. After staining, cells were sorted using a BD FACSAria IIIu cell sorter (BD Biosciences, San Jose, CA, USA).

### Proliferation assays

Proliferation assays were conducted using either a coulter-counter as previously described [18] (2 × 10^4^ HUVEC cells/well and 1 × 10^4^ U87MG cells/well) or using the WST-1 assay (3 × 10^3^ cells/well) were seeded in complete growth medium. The WST-1 assay was performed according to the instructions of the vendor (Roche Diagnostics). The population doubling time was computed as the ln(2)/slope of the proliferation growth curve using GraphPad Prism 5 software (GraphPad Software, La Jolla, CA,USA). For colony formation assay, U87MG cells (300 cells/well) were seeded in complete growth medium (10% FCS) and allowed to form colonies in 16 days. Colonies were fixed with PFA (4.0% v/v), stained with crystal violet (0.5% w/v) and photographed using an ImageQuant LAS-4000 machine (GE Healthcare, Chicago, IL, USA).

### Anchorage-independent colony formation assay

Colony formation in soft agar was performed as previously described [52]. The number of colonies and their average area were determined using the Image-pro premier morphometric software (Media Cybernetics, Rockville, MD, USA).

### Immunofluorescence

U87MG cells were plated on glass coverslips coated with fibronectin and actin fibers visualized as previously described [53]. The cells were imaged using a microscope (Inverted time-lapse phase microscope Observer Z1; Zeiss, Jena, Germany) equipped with a monochrome digital camera (AxioCam MRm; Zeiss) at 20x magnification. The images were acquired with ZEN lite software (Zeiss).

### Senescence associated β -Galactosidase assay

The staining of SA-β galactosidase in cells at pH-6 was performed using a senescence β-Galactosidase staining kit according to the instructions of the manufacturer (Cell signaling). Measurement of the absorbance at 420 nm from SA-β galactosidase activity detectable at pH 6.0 was performed as described [54].

### Cell cycle analysis

Cultured cells (1.5^.^10^6^ cells) were washed with PBS and trypsinized, then fixed in 70% ethanol at -20 °C for overnight. The cells were then incubated in a solution containing 0.1 mg/ml RNase (Thermo Fisher Scientific, Waltham, MA,USA) and 40 μg/ml propidium iodide (Sigma) at room temperature for 30 min, and finally cell cycle analysis was performed using flow cytometry (LSRFortessa; BD Biosciences) and FCS Express Flow Cytometry Software (De Novo Software, Pasadena, CA, USA).

### Analysis of mitotic spindle orientation

For the examination of spindle orientation in dividing cells Z-stack images were generated from 0.6 µm-thick optical sections using a LSM-880 Zeiss confocal microscope at 63x magnification. The images were acquired with ZEN lite software (Zeiss). Cells were immunostained with DAPI and antibodies targeting gamma-tubulin, alpha-tubulin antibodies. The linear distance and the vertical distance between the two poles of mitotic spindles was measured. Then, the angle between the axis of spindles and the cell-adhesion surface (α°, spindle angle) was calculated as described [35] using ZEN 2.3 software.

### Phosphorylation assays

The phosphorylation of p38 MAPK and AKT were determined as previously described for ERK1/2 phosphorylation [53] using western blot analysis. Quantification was performed using an ImageQuant Las-4000 machine (GE Healthcare).

### Tumor formation assays

The animal studies were all conducted according to the NIH guidelines and were approved by the Technion ethics committee. Power analysis was used to calculate the sample size required for animal experiments, and animals were single blind randomly divided into two different groups. U87MG control cells or U87MG cells silenced for plexin-A2 expression were washed, suspended in 100 µL PBS, and injected subcutaneously into 7-week-old female Athymic/Nude mice (2 × 10^6^ cells/mouse) or alternatively injected subcutaneously into 15-week-old male NOD/SCID mice (3 × 10^6^ cells/mouse) as indicated in the figure legends. Tumor development was measured twice a week using calipers. At the end tumors were excised and weighed.

### Sytox green nuclear staining

U87MG cells expressing a non-specific shRNA (ShC) or cells that were silenced for plexin-A2 expression were incubated with Sytox green nuclear staining (Invitrogen) for 15 min. Fluorescent nuclei of dead cells were detected using flow cytometry (LSRFortessa; BD Biosciences).

### Production, purification, and concentration of recombinant sema6A-fc fusion protein

A plasmid containing the extra-cellular region of sema6A fused to the human Fc was kindly received from Dr. Avraham Yaron (Weizmann Institute of Science, Rehovot, Israel). HEK293-FT cells were transfected with this plasmid using standard protocol and cultured with serum-free DMEM media. Conditioned media was collected 48 hr later and used in binding assays. Sema6a-Fc conditioned medium, was concentrated 20-fold using Amicon™ 30 K centrifuge filters.

### Alkaline phosphates binding Assay

U87MG KO clone 54.3 cells infected with a lentiviral vector directing expression of plexin-A2 (PlexA2) or with a variant containing a point mutation in the plexin-A2 semaphorin binding site (PlexA2/A396E). The cells were washed with binding buffer (Hank’s-Buffered Salt Solution with 0.5% BSA, 0.1% NaN3, 5 mM CaCl2, and 1 mM MgCl2 and 20 mM HEPES, pH-7.0) for 15 min and incubated for 45 min at 4 °C with 20-fold concentrated sema6A-Fc conditioned medium containing a 1/5000 fold diluted goat anti-human IgG antibody conjugated with alkaline phosphates (AP) (Jackson ImmunoResearch Laboratories). After the removal of unbound ligand, cells were fixed with 4% PFA for 20 min, and rinsed with 20 mM HEPES pH-7.0, 150 mM NaCl. To destroy intrinsic AP activity, cells were heat inactivated in 65 °C for 30 min. Finally, cells were incubated with 5-bromo-4-chloro-3-indolyl phosphate/ nitro blue tetrazolium chloride (BCIP/NBT) liquid substrate (Sigma-Aldrich (B1911)). After an hour cells were photographed with a phase-contrast inverted microscope (Eclipse TE2000-U; Nikon, Tokyo, Japan). Equipped with a digital photomicrographic camera (DXM1200C; Nikon) at 10x magnification. The images were acquired with ACT-1C software (Nikon). The mean area of stained cells relative to the mean area of total cells per microscopic field was obtained from 38 microscopic fields using Image-pro premier software.

### Statistical analysis

Statistical analysis was carried out using GraphPad Prism software. Statistical significance was determined using the Mann-Whitney one-tailed non-parametric test in order to compare non-normally distributed sample groups with unsimilar variance. None of the samples or animals were excluded from the statistical analysis. Data from at least three independent experiments unless otherwise stated, are represented as mean ± SD. The following designations were used: *P < 0.05, **P < 0.01 and ***P < 0.001 and nonspecific: ns. N represents the number of independent experiments. At least three replicates were done in each independent parameters measured in each experiment. No randomization method was followed to allocate samples to the various experimental groups. No predictive statistical methods were used to predetermine sample sizes. However, we adopted sample sizes (indicated in the figure legends) in the same range as those previously reported in the literature for similar experiments. Investigators were blinded when assessing the outcome of the experiments.

## Supplementary information

Supplementary Figure legends

Supplemental Figure 1

Supplemental Figure 2

Supplemental Figure 3

Supplemental Figure 4

Supplemental Figure 5

Supplemental Figure 6

Supplemental Figure 7

Supplemental Figure 8

Original Data File

Reproducibility checklist

## Data Availability

The data used and/or analyzed during the current study and the cell lines generated in the study are available from the corresponding author on reasonable request. Uncropped western blots can be seen in supplemental materials.
