# Supplementary material for: Plexin-A2 enables the proliferation and the development of tumors from glioblastoma derived cells
Source: Cell Death Dis. 2023 Jan 19;14(1):41. doi: 10.1038/s41419-023-05554-0 (PMC9852426; doi:10.1038/s41419-023-05554-0)
Supplement: Supplementary file 1 — Supplementary Figure legends [file 41419_2023_5554_MOESM1_ESM.doc]

Supplementary Figure legends

**Supp. Figure 1**

**Expression levels of plexin-A2 in human glioma and the effect of silencing plexin-A2 in HUVEC cells or in additional glioblastoma multiforme cells** **on their proliferation: (A)** Correlation of PlexinA2 mRNA expression with survival of GBM patients. Murat brain database, Oncomine [1, 2] **(B)** Effects of two shRNA species targeting plexin-A2 on the proliferation of human umbilical vein derived endothelial (HUVEC) cells. HUVEC cells expressing a non-specific shRNA (ShC) or HUVEC cells in which the expression of plexin-A2 was silenced using two shRNAs (ShPlexA2#1, or ShPlexA2#2) were seeded in triplicates (2x10^4^ cells/well) in 24 well plates in the presence or absence of bFGF (5 ng/ml). Adherent cells were counted in coulter counter after 3 days. Shown is the percentage of cells in day 3 as compared to the number of cells expressing a control shRNA (ShC) taken as 100%. Data are represented as mean ± SD. Statistical analysis was done using the one tailed Mann-Whitney test. N=4 ***P<0.001, **P<0.01, A western blots prepared from lysates of these cells were probed with an antibody directed against plexin-A2 or vinculin are shown in the upper panel. **(C)** Lentiviruses were used to express control (ShC) or a plexin-A2-targeting shRNA (ShA2#1) in U118, T98G and U373 cells. The expression of plexin-A2 was then examined using qRT-PCR. **(D)** U118, T98G and U373 cells were infected with lentiviruses encoding a control shRNA (ShC) or an shRNA targeting plexinA2 (ShPlexA2#1). Each group of cells was seeded in quadruplicate in 96 well dishes (3x10^3^ cells/well). Cell proliferation was measured using the WST-1 proliferation assay as described in materials and methods, and the values presented were calculated as described in Fig 1B. Results were derived from five (for U118 cells) or four (for T98G cells and U373 cells) independent experiments. Data are represented as mean ± SD. Statistical analysis was done using the one tailed Mann-Whitney test. Ns: non-specific. (**E**) Haematoxylin-eosin stained histological sections of tumors derived from U87MG expressing a non-specific shRNA (ShC) or cells silenced for plexin-A2 expression using ShPlexA2#1 and injected subcutaneously into NOD/SCID mice (3x10^6^ cells/mouse). (**F**) Second experiment in which U87MG expressing a non-specific shRNA (ShC) or cells that were silenced for plexin-A2 expression using ShPlexA2#1 were injected subcutaneously into Athymic/Nude mice (2x10^6^ cells/mouse). Each group contained 7 mice. Tumor development was measured twice a week using calipers. Data are represented as mean ± SD. **(G)** Western blots prepared from cell lysates of U87MG, A172, T98G, U118 and U373 glioblastoma cells. Blots were probed with antibodies directed against plexin-D1, plexin-A2 or plexin-A4. Antibodies directed against vinculin were used to compare loading.

**Supp. Figure 2**

**Silencing plexin-A2 in U87MG cells isn’t associated with increased cell death:** Cyto-fluorimetric analysis of SYTOX Green-stained U87MG expressing a non-specific shRNA (ShC) or cells that were silenced for plexin-A2 expression (ShPlexA2#1). P3 represents the percentage of dead cells.

**Supp. Figure 3**

**A dominant negative form of plexin-A2 lacking the intracellular domain (A2ExTm) inhibits the proliferation of U87MG cells: (A)** U87MG cells were infected with an empty lentiviral expression vector (U87MG+EV) or with a lentiviral vector directing expression of A2ExTm (U87MG+A2ExTm). Western blots prepared from cell lysates were probed with antibodies directed against plexin-A2 or actin**(B)** U87MG cells infected with an empty lentiviral expression vector (EV) or with a lentiviral vector directing expression of A2ExTm were seeded in 96 well dishes) 3x10^3^ cells/well). Cell proliferation was measured using the WST-1 proliferation assay as described in methods. Data are represented as mean ± SD. **(C)** The average population doubling time of the cells shown in A. Data are represented as mean ± SD. Statistical analysis was done using the one tailed Mann-Whitney test. *P<0.05, N=3 .

**Supp. Figure 4**

**Guide RNAs used for the generation of plexin-A2 knock-out U87MG cells: (A)** Sequences of the sgRNAs encoding primers used for CRISPR/Cas9 mediated knock-out of the plexin-A2 gene in U87MG cells. **(B)** Sequence analysis of two plexin-A2 knock-out U87MG clones of cells. Shown are the target sequences in the plexin-A2 gene and the frame shift mutations that were introduced into the two alleles of the plexin-A2 gene in knock-out clones 54 and 35 which were determined by sequencing and analyzed as described in materials and methods.

**Supp. Figure 5**

**U87MG cells silenced for plexin-A2 expression acquire properties of senescent cells and have altered mitotic spindle orientation additionally, in plexin-A2 knock-out U87MG cells, clone 35, the phosphorylation of AKT is inhibited and that of p38 induced:** **(A)** U87MG cells expressing a non-specific shRNA (ShC) or U87MG cells in which the expression of plexin-A2 was silenced using a shRNA (ShPlexA2#1) were assayed at pH-6 for the expression of the senescence marker SA-β galactosidase. **(B)** Shown are 3D confocal photographs generated using the Imaris software of the indicated cell types. Staining was performed as described in Fig. 6A. Scale bars: 10 μm . **(C)** The phosphorylation levels of AKT were assayed by western blot analysis of cell lysates as described in methods, using an antibody directed against phosphorylated AKT (ser473). Loading was assessed using an antibody directed against total AKT. Shown is a representative western blot. The effect of plexin-A2 knock-out on the average phosphorylation levels of AKT was determined in four independent experiments. Below is shown a histogram depicting the average ratio between the intensity of the respective phospho-AKT bands and the total AKT bands. Data are represented as mean ± SD. Statistical analysis was done using the one tailed Mann-Whitney test. Ns: non-specific, **(D)** The phosphorylation levels of p38 were assayed as described in methods, using an antibody directed against phosphorylated p38 (Thr180/Tyr182). Loading was assessed using an antibody directed against total p38. Shown is a representative western blot. Shown is a representative western blot. The effect of plexin-A2 knock-out on the average phosphorylation levels of p38 was determined in four independent experiments. Below is shown a histogram depicting the average ratio between the intensity of the respective phospho-p38 bands and the total p38 bands. Data are represented as mean ± SD. Statistical analysis was done using the one tailed Mann-Whitney test. *P<0.05.

**Supp. Figure 6**

**Generation of A2InTm and full length plexin-A2 variants containing point mutations: (A)** Sequences of point mutations introduced into the A2InTm cDNA encoding the intracellular and trans-membrane domain of plexin-A2 **(B)** Western blots prepared from cell lysates of clone 54.3 knock-out cells (54.3) expressing cDNAs encoding an empty expression vector (EV), a V5 tagged A2InTm or A2InTm variants containing point mutations in the indicated subdomains of the plexin-A2 intracellular domain were probed with antibodies directed against the V5 epitope tag of A2InTm or vinculin. (**C**) Sequences of a point mutation introduced into plexin-A2 cDNA at the semaphorin binding site. (**D**) Western blots prepared from cell lysates of clone 54.3 knock-out cells (54.3) expressing cDNAs encoding an empty expression vector (EV), a full length plexin-A2 (54.3+PlexA2) or a plexin-A2 variant mutated at the semaphorin binding site (54.3+A396E) were probed with antibodies directed against plexin-A2 or actin. **(E)** Western blots prepared from cell lysates of clone 54.3 knock-out cells (54.3) expressing cDNAs encoding an empty expression vector (EV), a V5 tagged A2InTm (54.3+A2InTm) or an A2InTm variant mutated at the conserved catalytic arginine residues of the GAP domain (54.3+ R1428/1429A) were probed with antibodies directed against the V5 epitope tag of A2InTm or actin.

**Supp. Figure 7**

**A point mutation in the semaphorin binding site of plexin-A2 inhibits sema6A binding: (A)** Clone 54.3 knock-out cells (54.3) expressing cDNAs encoding a full length plexin-A2 (Clone 54.3+PlexA2) or cells expressing a plexin-A2 variant mutated at the semaphorin binding site (Clone 54.3+PlexA2/A396E) were incubated with 20x fold concentrated conditioned medium derived from HEK293 cells expressing the cDNA encoding sema6A-Fc that contained in addition diluted (1/5000) goat anti-human IgG alkaline phosphates (AP) conjugated antibody. Alkaline phosphatase staining was performed as described in materials and methods. Stained cells were photographed as described. Shown are representative photographs. **(B)** The mean area of stained cells relative to the mean area of total cells per microscopic field was obtained from N microscopic fields obtained for each cell type using Image-pro premier software. Data are represented as mean ± SD. Statistical analysis was done using the one tailed Mann-Whitney test. ***P<0.001, (Clone 54.3+PlexA2 N=16), (Clone 54.3+PlexA2/A396E N=22).

**Supp. Figure 8**

**The pro-proliferative effects of plexin-A2 are not induced by sema3C: (A)** Conditioned medium from U87MG cells or from HEK293 cells over-expressing sema3C [3] that were seeded at equal concentrations was concentrated 20-fold by centrifugation using Amicon™ 30K centrifuge filters. Shown is a western blot probed with an antibody directed against sema3C. Coomassie blue staining of the gel is shown as a loading control. **(B)** U87MG cells infected with an empty lentiviral expression vector (U87MG+EV) as well as U87MG cells expressing recombinant plexin-D1 (U87MG+Plexin-D1) [4], were stimulated with elution buffer (Control) or with 1 µg/ml purified sema3E/Fc [5]. Following a 30 min. incubation at 37^o^C the cells were photographed. **(C)** Representative growth curves of U87MG cells and of two independent U87MG derived clones of cells in which both the NRP1 and NRP2 genes were knocked-out [4]. Cells were seeded (1x10^4^ cells/well) and adherent cells were counted every two days using a coulter-counter. Data are represented as mean ± SD. **(D)** The average population doubling times of the cell types shown under C. Data are represented as mean ± SD. Statistical analysis was done using the one tailed Mann-Whitney test. N=5 ns: non-specific.

References

1. Murat A, Migliavacca E, Gorlia T, Lambiv WL, Shay T, Hamou MF et al. Stem cell-related "self-renewal" signature and high epidermal growth factor receptor expression associated with resistance to concomitant chemoradiotherapy in glioblastoma. J Clin Oncol. 2008;26:3015-24.

2. Man J, Shoemake J, Zhou W, Fang X, Wu Q, Rizzo A et al. Sema3C promotes the survival and tumorigenicity of glioma stem cells through Rac1 activation. Cell Rep. 2014;9:1812-26.

3. Mumblat Y, Kessler O, Ilan N, Neufeld G. Full length semaphorin-3C functions as an inhibitor of tumor lymphangiogenesis and tumor metastasis. Cancer Res. 2015;75:2177-86.

4. Smolkin T, Nir-Zvi I, Duvshani N, Mumblat Y, Kessler O, Neufeld G. Complexes of plexin-A4 and plexin-D1 convey semaphorin-3C signals to induce cytoskeletal collapse in the absence of neuropilins. J Cell Sci. 2018;131:

5. Casazza A, Kigel B, Maione F, Capparuccia L, Kessler O, Giraudo E et al. Tumour growth inhibition and anti-metastatic activity of a mutated furin-resistant Semaphorin 3E isoform. EMBO Mol Med. 2012;4:234-50.
