# Supplementary figures and images for: Plexin-A2 enables the proliferation and the development of tumors from glioblastoma derived cells
Source: Cell Death Dis. 2023 Jan 19;14(1):41. doi: 10.1038/s41419-023-05554-0 (PMC9852426; doi:10.1038/s41419-023-05554-0)

Supp. Figure 1

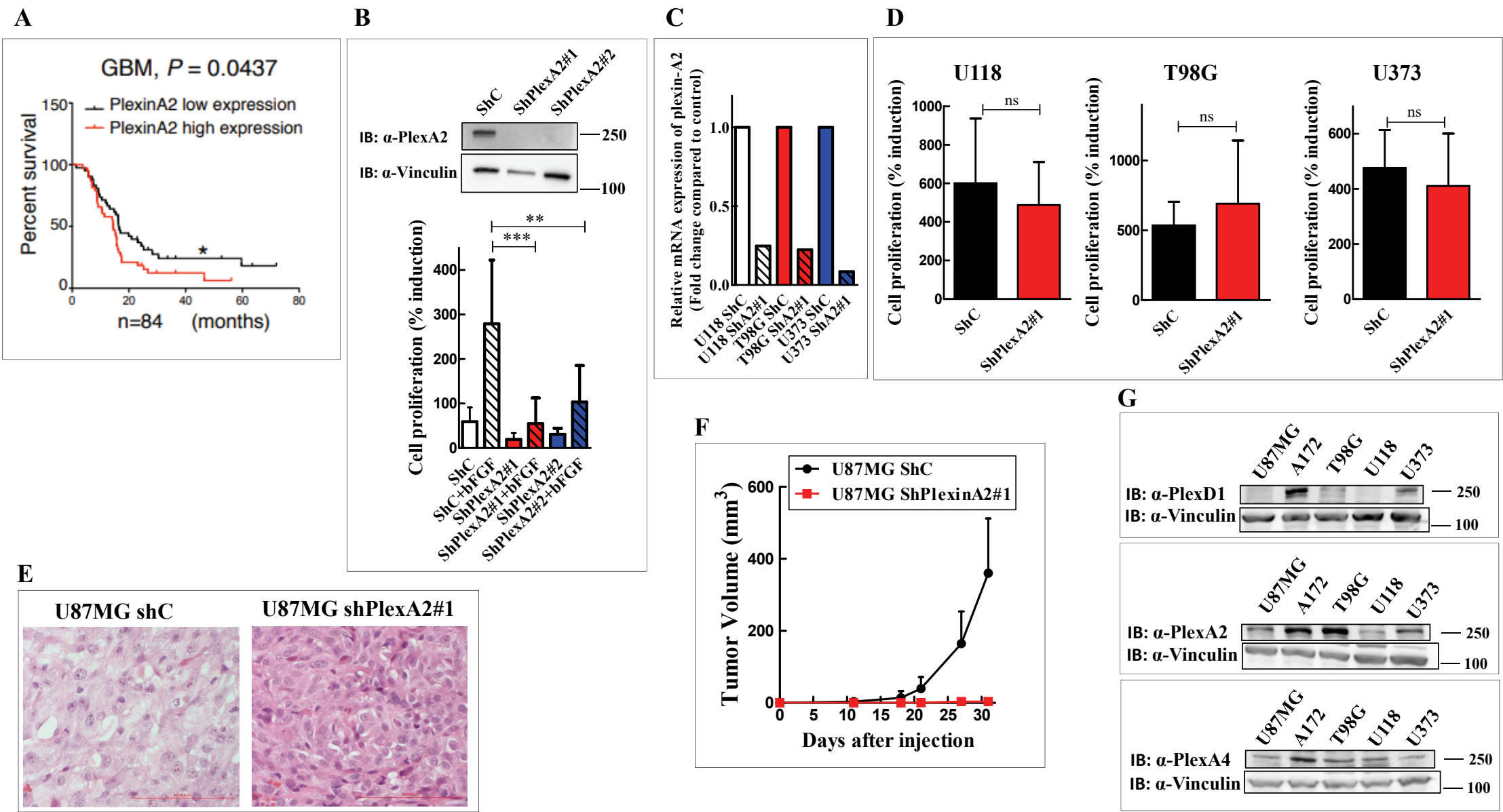

Supplement: Supplementary file 2 — Supplemental Figure 1 [file 41419_2023_5554_MOESM2_ESM.pdf]

Supp. Figure 2

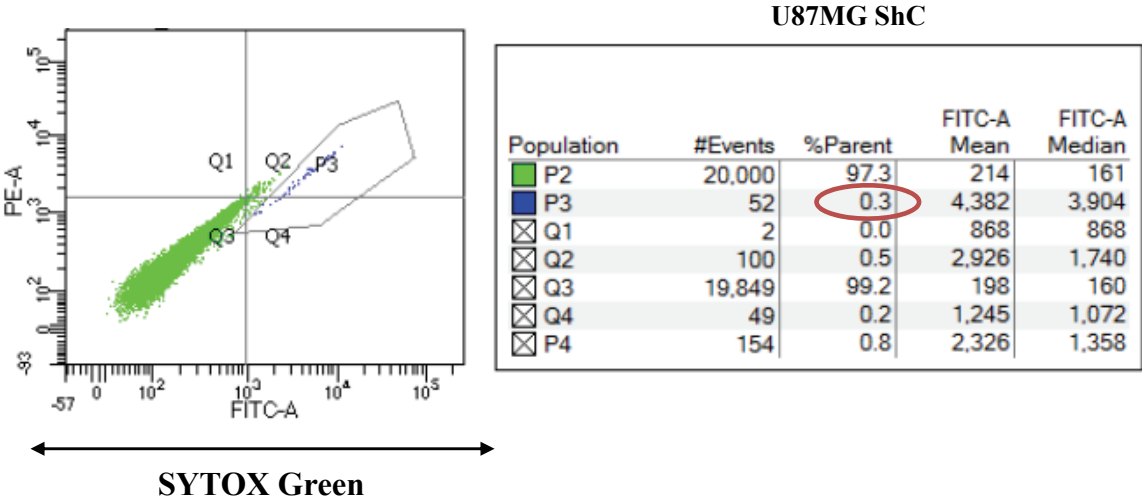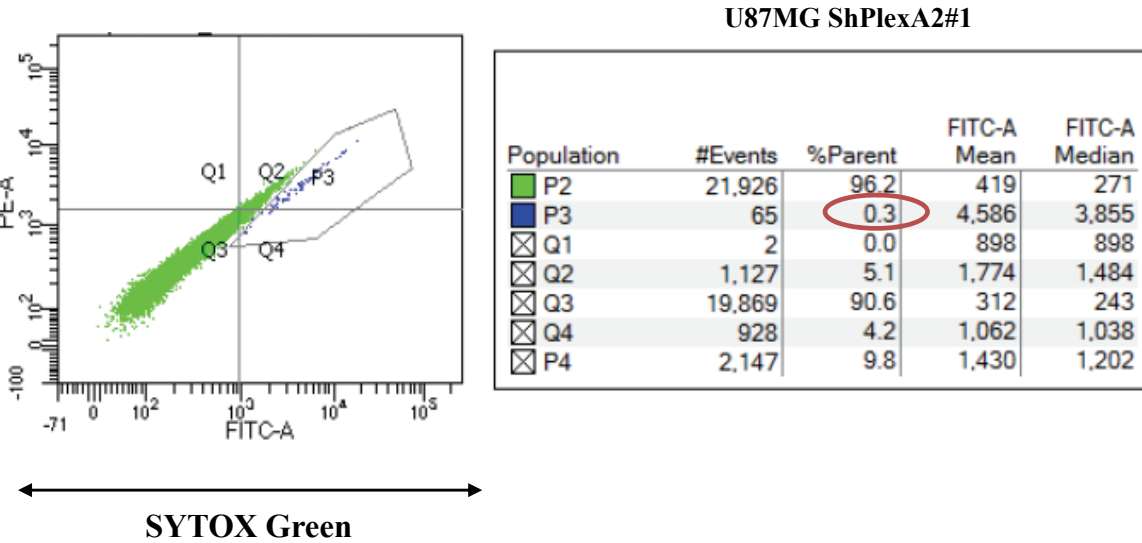

Supplement: Supplementary file 3 — Supplemental Figure 2 [file 41419_2023_5554_MOESM3_ESM.pdf]

Supp. Figure 3

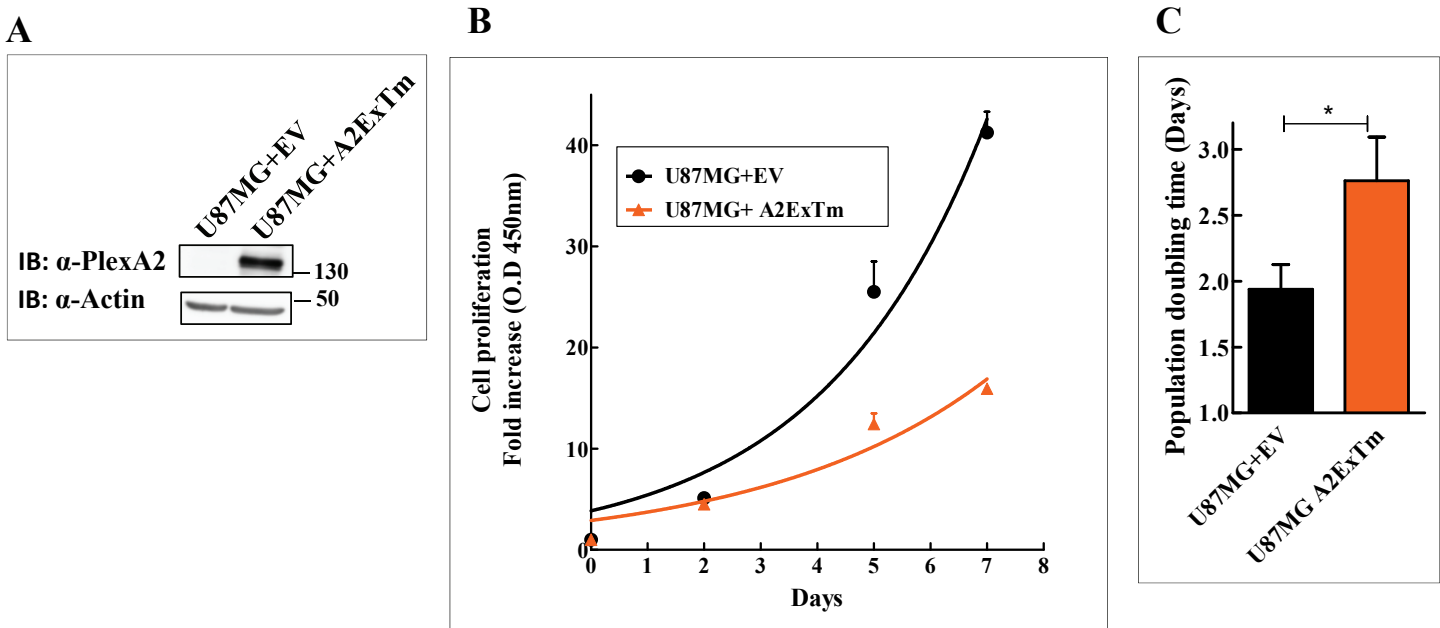

Supplement: Supplementary file 4 — Supplemental Figure 3 [file 41419_2023_5554_MOESM4_ESM.pdf]

Supp. Figure 5

A

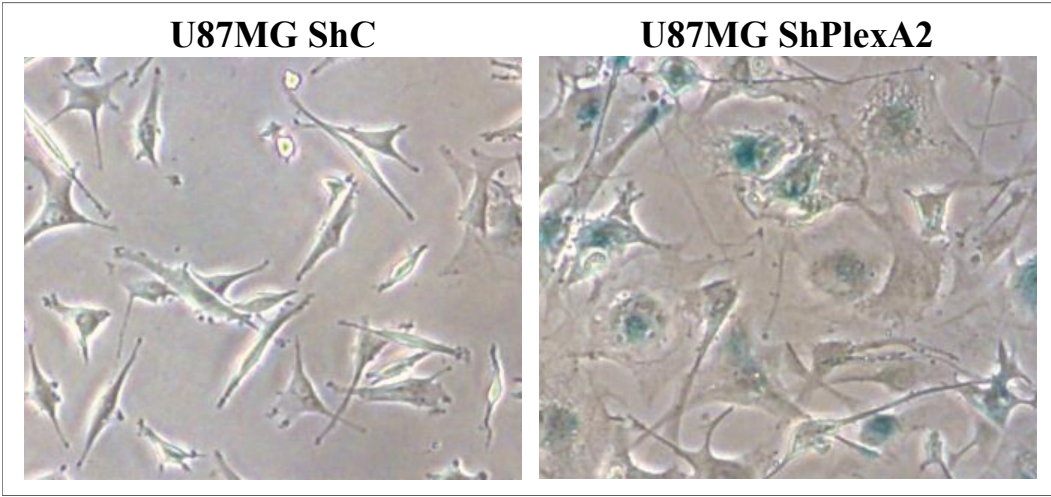

B

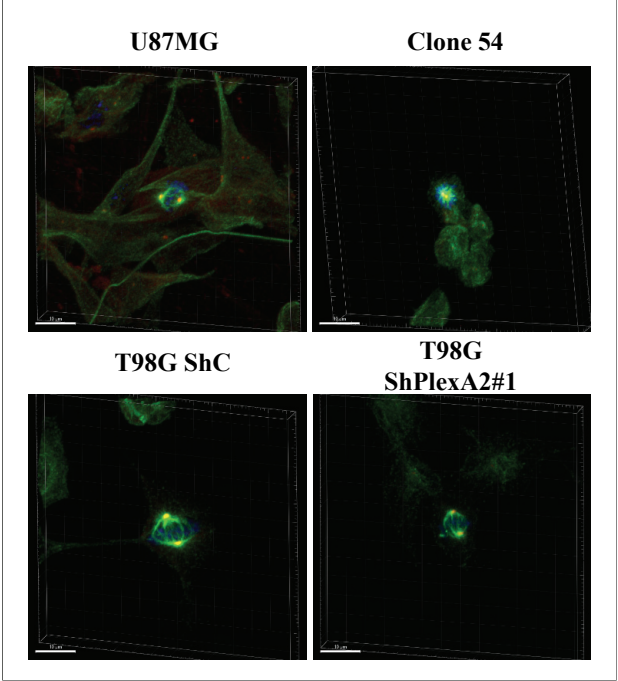

C

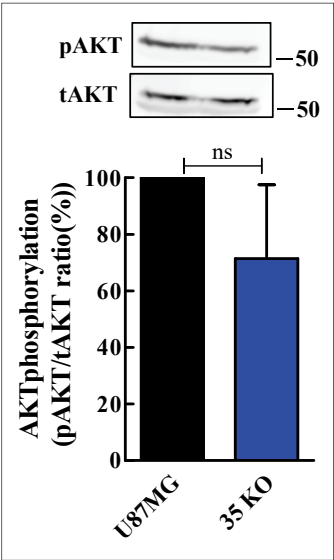

D

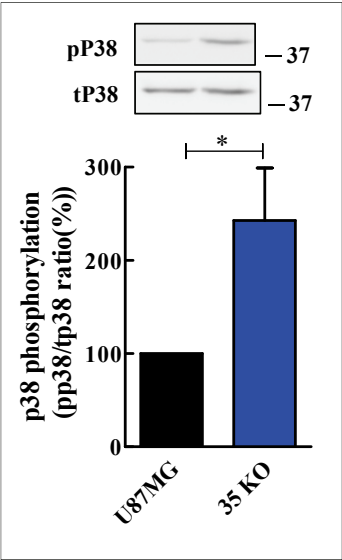

Supplement: Supplementary file 6 — Supplemental Figure 5 [file 41419_2023_5554_MOESM6_ESM.pdf]

Supp. Figure 6

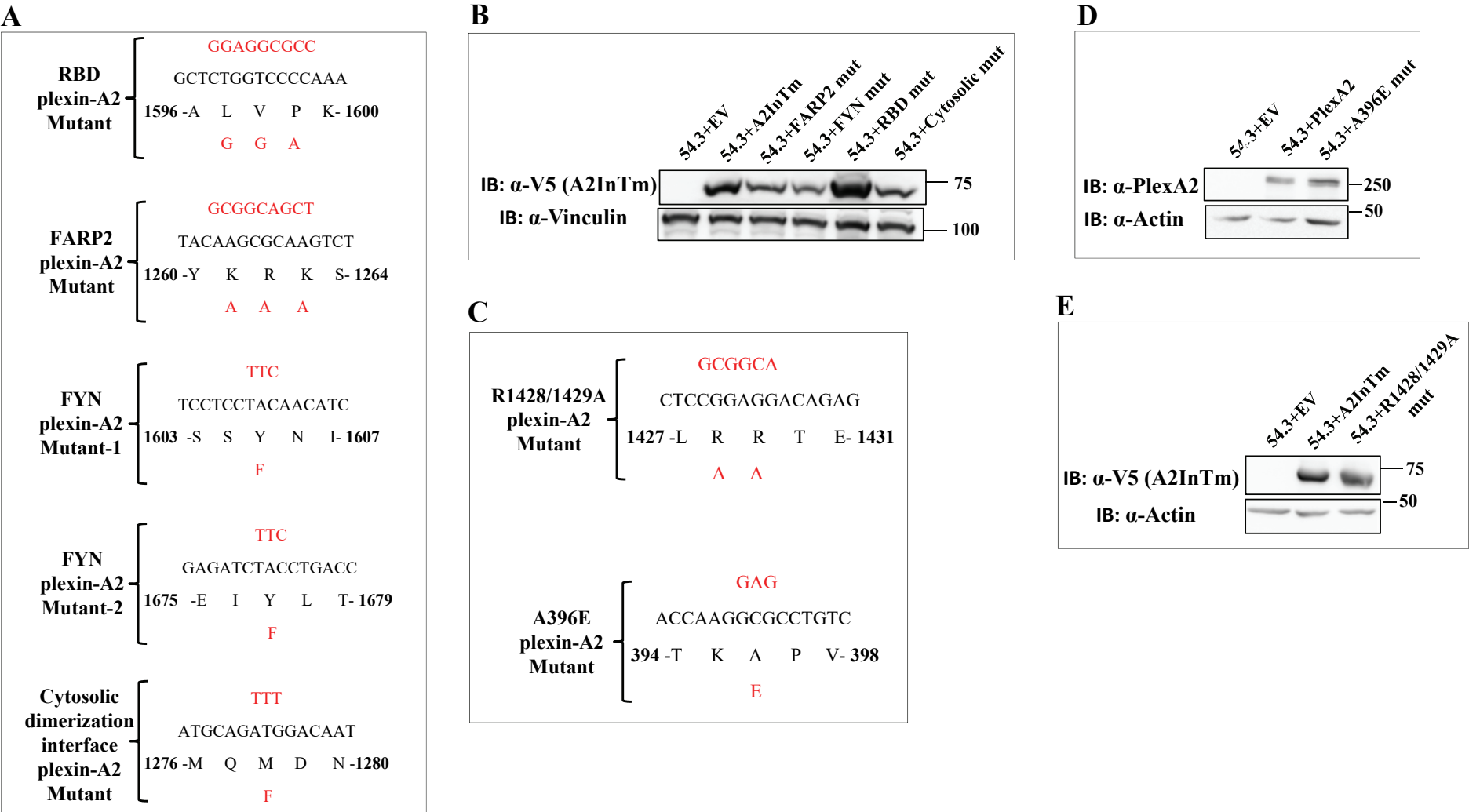

Supplement: Supplementary file 7 — Supplemental Figure 6 [file 41419_2023_5554_MOESM7_ESM.pdf]

Supp. Figure 7

A

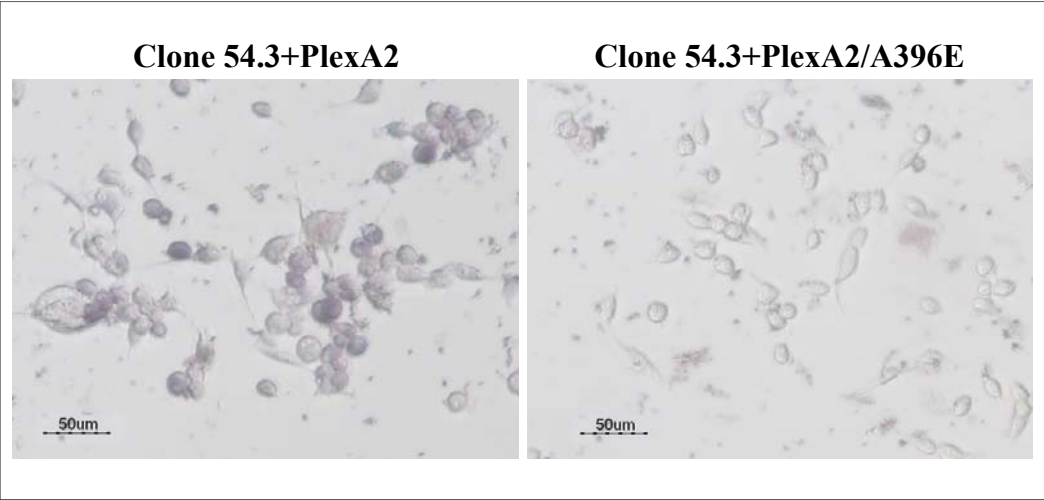

B

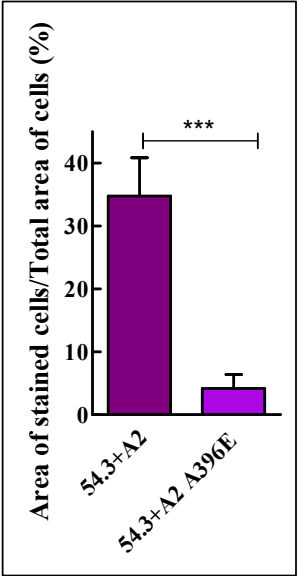

Supplement: Supplementary file 8 — Supplemental Figure 7 [file 41419_2023_5554_MOESM8_ESM.pdf]

Supp. Figure 8

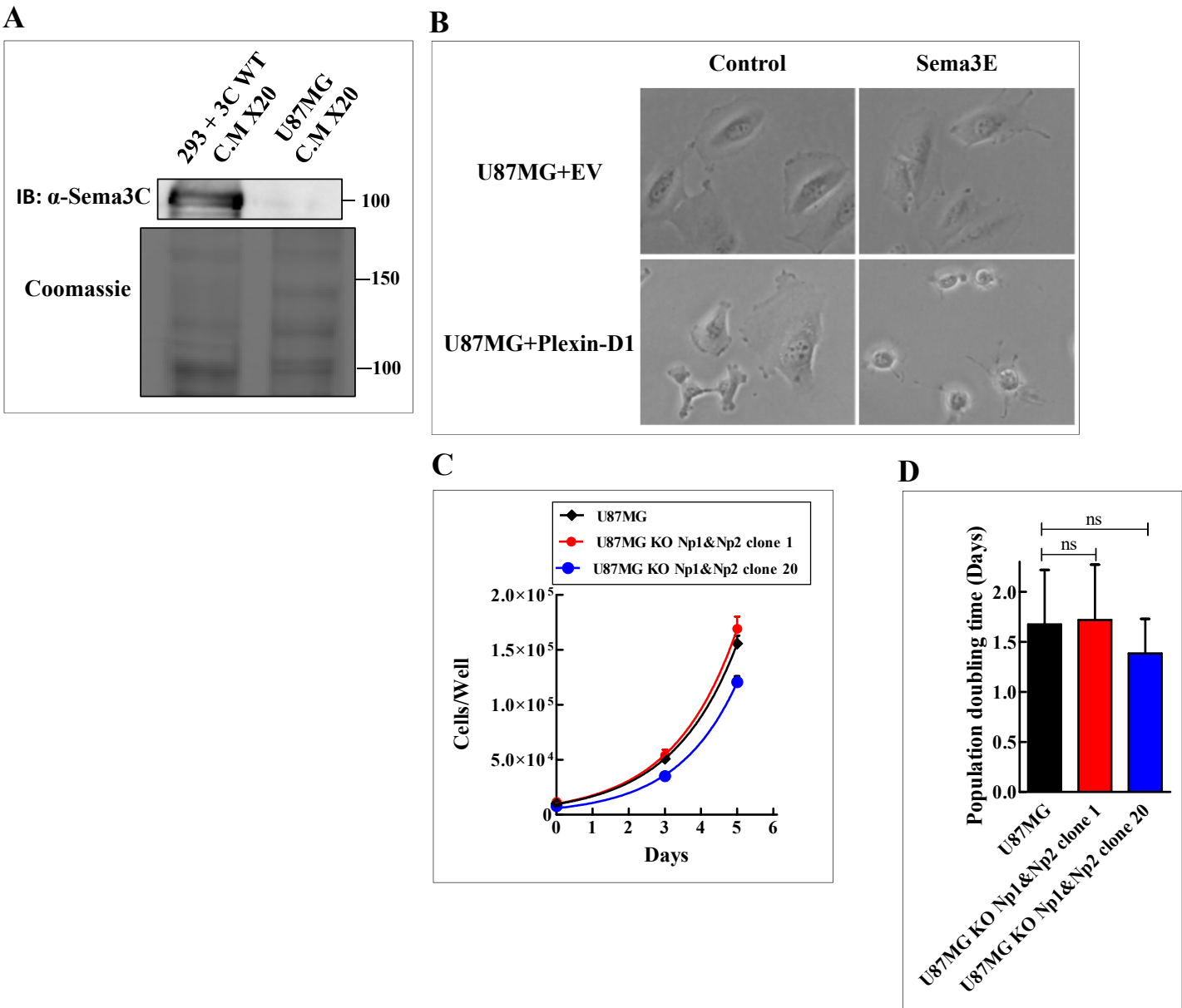

Supplement: Supplementary file 9 — Supplemental Figure 8 [file 41419_2023_5554_MOESM9_ESM.pdf]
