# Supplementary material for: Plexin-A2 enables the proliferation and the development of tumors from glioblastoma derived cells
Source: Cell Death Dis. 2023 Jan 19;14(1):41. doi: 10.1038/s41419-023-05554-0 (PMC9852426; doi:10.1038/s41419-023-05554-0)
Supplement: Supplementary file 5 — Supplemental Figure 4 [file 41419_2023_5554_MOESM5_ESM.pdf]

Supp. Figure 4

A

	**Top**	**Bottom**
**sgRNA1**	CACCGTCTACACCTCACGCATCGTG	AAACCACGATGCGTGAGGTGTAGAC
**sgRNA2**	CACCGGCAAGTTGATGGCCCGGATA	AAACTATCCGGGCCATCAACTTGCC

B

Changes generated by sgRNA1 to both alleles of clone 54

PAM ⬇

TACACCTCACGCATCGTGCGGCTCTGCAAGGATGACCCCAAGTTCCACTC — hPlexinA2 Consensus

TACACCTCACGCATCCGTGCGGCTCTGCAAGGATGACCCCAAGTTCCACTC — Allele 1: 1 bp insertion

TACACCTCACGCATGGCGTGCGGCTCTGCAAGGATGACCCCAAGTTCCACTC — Allele 2: 2 bp insertion

Changes generated by sgRNA2 to both alleles of clone 35

PAM ⬇

TCTGCCCTGTGTGCCTTCCCTATCCGGGCCATCAACTTGCAGATCAAGG — hPlexinA2 Consensus

TCTGCCCTGTGTGCCTTCCCTATCCGGGCCATCAACTTGCAGATCAAGG — Allele 1: 1 bp deletion

TCTGCCCTGTGTGCCTTCCCTATCCAAGGGCCATCAACTTGCAGATCAAGG — Allele 2: 1 bp insertion
