# Supplementary material for: Plexin-A2 enables the proliferation and the development of tumors from glioblastoma derived cells
Source: Cell Death Dis. 2023 Jan 19;14(1):41. doi: 10.1038/s41419-023-05554-0 (PMC9852426; doi:10.1038/s41419-023-05554-0)
Supplement: Supplementary file 10 — Original Data File [file 41419_2023_5554_MOESM10_ESM.doc]

Supplementary original western blots

**Figure 1**

**Figure 2**

**Figure 3**

**Figure 4**

**Figure 6**

**Figure 7**

**Supp. Figure 1**

**Supp. Figure 3**

**Supp. Figure 5**

**Supp. Figure 6**

**Supp. Figure 8**
